# MYC/MAX balance dictates cell progenitor fate by altering the HOX program in the *Drosophila* eye

**DOI:** 10.3389/fcell.2026.1958701

**Published:** 2026-10-26

**Authors:** Sara Monticelli, Giorgio Milazzo, Suleman Khan Zadran, Martina Santulli, Nicola Balboni, Silvia Strocchi, Ettore De Giorgio, Pieter Mestdagh, Angela Giangrande, Roberto Bernardoni, Giovanni Perini

**Affiliations:** 1 Department of Pharmacy and Biotechnology, Alma Mater Studiorum - University of Bologna, Bologna, Italy; 2 University of Ghent, Campus UZ, Ghent, Belgium; 3 IGBMC, Institut de Génétique et de Biologie Moléculaire et Cellulaire, Illkirch, France; 4 Centre National de la Recherche Scientifique, Illkirch, France; 5 Institut National de la Santé et de la Recherche Médicale, Illkirch, France; 6 Université de Strasbourg, Illkirch, France; 7 Istituto di Ricerca e Cura a Carattere Scientifico (IRCCS) AOUBO Sant’Orsola - Laboratory of Preclinical and Translational Research in Oncology (PRO), Bologna, Italy

**Keywords:** cell transdetermination, *Drosophila*, hox genes, MYC, MYC/MAX ratio

## Abstract

The transcription factor MYC acts canonically as heterodimer with MAX to promote growth, proliferation, and metabolic adaptation. Although MYC and MAX relative levels are normally kept in balance under physiological conditions, those levels are often dysregulated in many human cancers where MYC is overexpressed and MAX can sometimes be reduced. How altered stoichiometry of MYC/MAX can impact on cell biology is not completely understood. Using the *Drosophila* eye amenable to genetic modifications, we demonstrate that the relative abundance of the fly MYC ortholog Myc and its partner Max affect progenitor cell fate. Elevating the Myc/Max ratio—through Myc or human MYCN overexpression, Max depletion, or combined manipulations—disrupts photoreceptor differentiation and ectopically induces the thoracic HOX gene *Antennapedia* within the eye disc. When such a ratio is driven to an extreme degree, the change in cell fate results in eye-to-wing transdetermination. Conversely, restoring Max levels rescues these phenotypes, demonstrating that stoichiometry, not absolute MYC levels, shapes the response. Transcriptomic profiling reveals global repression of eye-specific regulators (*e.g., eyegone* and *prospero*) and ectopic activation of wing determinants (*e.g., vestigial* and *nubbin*) while genetic interactions identify the cephalic HOX gene *Deformed* as a key mediator modulating MYC-dependent identity changes. Our findings uncover MYC/MAX imbalance as a developmental switch that can rewire HOX expression and reprogram progenitor identity, suggesting a conserved mechanism underlying MYC-driven cellular plasticity in cancer.

## Graphical Abstract

**Figure:**
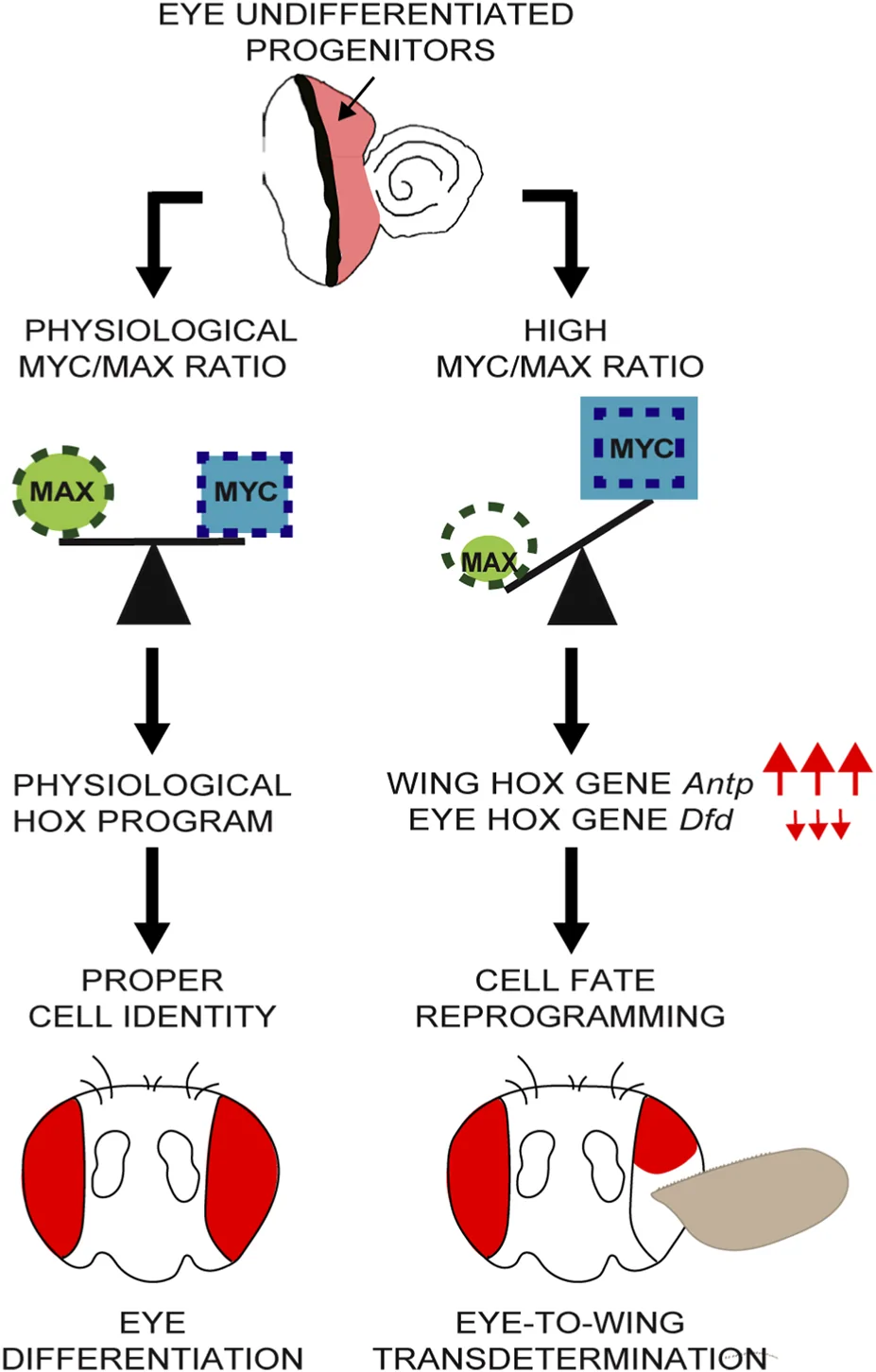

## Introduction

The MYC family of transcription factors (MYC, MYCN, and MYCL) modulates essential cellular processes, including growth, proliferation, metabolism, apoptosis and differentiation, by controlling a broad array of target genes. Dysregulation of MYC proteins, and consequently of these processes, contributes to the initiation and progression of numerous human cancers (Dhanasekaran et al., 2022). Intriguingly, recent evidence highlights a role for MYC proteins in tumor cell transdifferentiation, the direct conversion of one differentiated cell type into another, which contributes to tumor heterogeneity and therapy resistance (Patel et al., 2021; Quintanal-Villalonga et al., 2021). Although the precise mechanisms underlying MYC-mediated control of cell fate remain elusive, MYC-driven chromatin reshaping can disrupt the normal expression of HOX genes (Kress et al., 2015), key developmental regulators of cell identity that are frequently deregulated in cancer (Yadav et al., 2024).

In the canonical model, MYC proteins heterodimerize with MAX, forming the transcriptionally active complex that regulates target gene expression, making MYC activity generally MAX-dependent (Dhanasekaran et al., 2022)**.** Human cancers overexpress MYC factors at varying degrees and through distinct genetic mechanisms including chromosomal translocations, genomic amplification and promoter mutations (Dhanasekaran et al., 2022). A striking example is childhood neuroblastoma, in which MYCN is amplified to hundreds of copies (Schaub et al., 2018). This implies that upon MYC overexpression, MAX levels should not represent a limiting factor. However, large deletions or loss of function mutations in the MAX gene have been identified in a subset of tumors (Bausch et al., 2017), suggesting that MAX-independent functions of MYC also contribute to tumorigenesis. In line with that, the most aggressive neuroblastomas are those in which MYCN is overexpressed while MAX levels are below normal (Ferrucci et al., 2018). Recent work has also shown that MYCN modulates RNA metabolism upon recruiting the nuclear exosome, a 3′-5′ exoribonuclease complex, to its target genes and binding intronic transcripts, thus facilitating S-phase progression and enhancing stress tolerance of neuroblastoma cells (Papadopoulos et al., 2022; Papadopoulos et al., 2024). These functions do not seem to directly require MAX and call for deeper investigation into how MYCN, and more broadly MYC proteins, influence cellular behavior and tumor progression outside of their dimerization with MAX**.**

To dissect oncogenic mechanisms controlled by MYC and/or MAX *in vivo*, it is essential to study the effects of altering their absolute and relative expression levels. The fruit fly, *Drosophila melanogaster*, provides an ideal system for this purpose, as it carries single orthologs (Myc and Max) and sophisticated genetic tools to constitutively or conditionally modulate gene expression are available. Many functions of the vertebrate MYC are conserved in flies (Grifoni and Bellosta, 2015), including regulation of cell growth (Secombe et al., 2007; Steiger et al., 2008) and, during physiological cell competition, promotion of cell proliferation (Cova et al., 2004; Moreno and Basler, 2004). Here, we demonstrate that a high Myc/Max ratio in undifferentiated eye progenitors disrupts cell differentiation and promotes eye-to-wing transdetermination by altering the physiological expression of HOX genes**.** Genetic and transcriptomic analyses reveal ectopic activation of the wing HOX gene *Antennapedia* and the wing developmental program. This is enhanced by loss of the HOX factor Deformed and accompanied by suppression of the eye developmental program. Our findings offer a novel perspective on MYC’s oncogenic role, highlighting cell fate reprogramming as a crucial mechanism through which MYC may influence tumor progression.

## Materials and methods

### Fly lines

Flies were reared on standard corn-yeast medium at 22 °C. The following strains were used: *ey-Gal4* (Betz et al., 2001)*, UAS-dGFP* (gift from Gary Struhl) (Lieber et al., 2011), *UAS-Myc* (BDSC #9674), *Max-RNAi* (BDSC #29328), *Dfd-RNAi* (BDSC #50792), *GFP-RNAi* (BDSC #41553 and BDSC #41555), *Myc* ^*G0354*^ (BDSC #11981), *Max* ^*1*^ (gift from Peter Gallant) (Steiger et al., 2008), *Max-rescue* (gift from Peter Gallant) (Steiger et al., 2008), *HDAC1-RNAi* (BDSC #33725), *HDAC1* ^*04556*^ (BDSC #11633), *Smr-RNAi* (BDSC #27068), *Smr* ^*G0060*^ (BDSC #11653). *UAS-MYCN* was generously donated by Daniela Grifoni (unpublished, daniela.grifoni@univaq.it). Unless otherwise specified, both male and female animals were used in this study, to avoid gender bias.

### Phenotypic analysis of adult eyes

Animals were raised at the indicated temperatures (24 °C or 29 °C), and young adults (1–3 days old) were used for phenotypic analysis. Adult eyes were examined using a stereomicroscope to score for eye defects. For each genotype and temperature, > 100 flies were analyzed. Individual animals were considered as the experimental unit. For all experiments, animals were collected from at least three independent vials issued, when possible, from independent crosses to ensure that the observed phenotypic distributions were reproducible across independent populations. The adult eye phenotypes were classified into ordered categories reflecting increasing phenotypic severity (high-Myc, low-Max and high-Myc-low-Max categories, see Results). Phenotypic comparisons between experimental and control groups were performed using the Mann-Whitney U test as an appropriate non-parametric test to compare the distributions of these ordinal phenotypic scores between two groups.

### Fluorescent immunolabeling on eye imaginal discs

Wandering third instar larvae (wL3) were collected ~120 h after egg lay at 24 °C or ~91 h at 29 °C from eggs laid on yeast-supplemented agar medium. Larvae were dissected to expose the eye-antenna imaginal complex, fixed in 4% paraformaldehyde (Electron Microscopy Sciences, Cat# 50-980-487) in 1× PBS for 20 min, and permeabilized in 0.3% Triton X-100 in 1× PBS (0.3% PBT) for 1 h. Samples were blocked in 4% Normal Goat Serum (Jackson Immunoresearch, RRID AB_2336990) in 0.3% PBT for 20 min and incubated overnight at 4 °C with primary antibodies diluted in blocking solution. The following primary antibodies were used: monoclonal rat anti-Elav (DSHB 7E8A10, RRID AB_5282. 1:100), monoclonal mouse anti-Antp (DSHB 4C3, RRID AB_528082, 1:400), rabbit anti-Phospho Histone H3 (Upstate Biotechnology, RRID AB_310177, 1:100), mouse anti-Nubbin (DSHB 2D4, RRID AB_2722119, 1:50). The next day, larvae were washed in 0.3% PBT, blocked for 20 min, and incubated with secondary antibodies for 2 h. The following secondary antibodies were used: anti-mouse Cy3 (Jackson Immunoresearch, RRID AB_2340813, 1:400), anti-rat FITC (Jackson Immunoresearch, RRID AB_2340632, 1:400), anti-rabbit Cy5 (Jackson Immunoresearch, RRID AB_2340607, 1:400). All the secondary antibodies were subtracted against the other hosts species to avoid cross-reactions. Cell nuclei were labelled with 2 μg/mL HOECHST 33258 (Sigma-Aldrich, Cat# 94403) in 1X PBS for 5 min. After three washes in 0.3% PBT, discs were isolated, mounted in Fluoromount-G (Electron Microscopy Sciences, Cat# 17984-25), dried overnight at room temperature, and imaged or stored at −20 °C.

Phenotypic analyses of immunolabeled eye discs were based on ≥15 discs from 2 to 3 independent immunolabeling experiments.

### Imaging

Bright-field images of adult eyes and imago adults were captured as Z-stacks using a Nikon Eclipse T90i microscope at ×10 magnification. Stacks were processed into single focused images using the Enhanced Deep Focus (EDF) module of NIS-Elements AR 3.10 software (Nikon).

Immunolabeled imaginal discs were imaged as Z-stacks with a Nikon Eclipse Ti2/A1R confocal microscope equipped with a PlanApo ×40 objective and captured using NIS-Elements AR 3.10. Maximum intensity projections generated in NIS-Elements AR 3.10 are shown in the figures, unless otherwise specified.

### Real-time quantitative PCR

Eye imaginal discs, separated from antennal discs, were dissected from 15 wL3 male larvae. Total RNA was extracted using TRI Reagent® (Sigma-Aldrich, Cat# T9424) according to the manufacturer’s instructions and resuspended in 15 µL RNase-free water. RNA was treated with the DNA-free™ Kit (Thermo Fisher, Cat# AM1906) and reverse-transcribed using iScript™ Reverse Transcription Supermix (Bio-Rad, Cat# 1708840) with random primers. Reverse transcription was performed at 25 °C for 5 min, 42 °C for 30 min, and 85 °C for 5 min. Gene expression was quantified by real-time quantitative PCR using SsoAdvanced™ SYBR® Green Supermix (Bio-Rad, Cat# 1725270) on a CFX96 real-time PCR system (Bio-Rad). The PCR program consisted of an initial denaturation at 95 °C for 30 s, followed by 30 cycles of 95 °C for 5 s and 60 °C for 30 s. A melting curve analysis was performed to verify amplification specificity. Relative expression of *Myc* and *Max* was calculated using the ΔΔCt method, with *RpL32n* and *Gapdh1* as reference genes. The primers used are listed below:

**Table:** 

​	Forward primer	Reverse primer
RpL32	TGC​TAA​GCT​GTC​GCA​CAA​ATG​G	TTC​GAT​CCG​TAA​CCG​ATG​TTG​G
Gapdh1	AAT​CAA​GGC​TAA​GGT​CGA​GGA​G	AAT​GGG​TGT​CGC​TGA​AGA​AGT​C
Myc	AAC​GAT​ATG​GTG​GAC​GAT​GGT​C	GGG​ATT​TGT​GGG​TAG​CTT​CTT​G
Max	GCG​GAA​GAT​CAG​TGA​AAA​CCA​G	TCC​CCA​TTT​GAA​CTC​TCC​AGT​G

### Bulk RNA-sequencing assay

Larvae of the indicated genotypes (*2x ey-Gal4, 2x UAS-Myc, 2x Max-RNAi* or *2x ey-Gal4, 4x GFP-RNAi)* were generated from 2 h egg lays on yeast-supplemented agar medium and reared at 24 °C until the wandering third instar (wL3) stage (~120 h after egg lay). For each genotype, at least 40 eye imaginal discs were dissected, separated from the antennal discs, and collected in TRI Reagent® (Sigma-Aldrich, Cat# T9424) for RNA extraction, following the manufacturer’s instructions. Three independent biological replicates were performed. mRNA libraries were prepared using the TruSeq RNA Library Prep Kit (Illumina, Cat# RS-122-2001) and sequenced in paired-end mode with 76 bp reads.

Transcript abundance was quantified with Salmon v0.12.0 (Patro et al., 2017) against the *Drosophila melanogaster* reference transcriptome (dm6/BDGP6.32), using the--validateMappings and--gcBias options to improve mapping accuracy and correct for GC-content bias. Statistical analyses were performed in R 4.2.0 with Bioconductor 3.15. Transcripts’ quantifications were imported using the tximeta package (Love et al., 2020), and differential expression was assessed with DESeq2 (Love et al., 2020). Log2 fold changes were shrunk using the Bayesian estimator apeglm (Zhu et al., 2019) to improve accuracy with limited replicates. P-values were adjusted for multiple testing using the Benjamini–Hochberg false discovery rate (FDR), with adjusted p < 0.05 considered significant. Normalized counts were obtained through DESeq2 internal normalization followed by rlog transformation. Gene Ontology enrichment was performed with PANGEA ((Hu et al., 2023) https://www.flyrnai.org/tools/pangea/web/home/7227) on differentially expressed genes (DEGs), defined as those with | log2FC(high-Myc-low-Max/control) | > 1, adjusted p < 0.05. baseMean ≥ 14 (25th percentile cutoff) was used to exclude lowly expressed genes. The GO biological processes set was used.

## Results

### Overexpressing fly Myc or human MYCN in eye progenitors affects eye formation

To investigate the impact of high MYC levels on cell fate, we used the fly eye as an *in vivo* model. The eye develops from an eye primordium (imaginal disc), that forms in the embryo and proliferates in the larva. At late larval stage (wandering third larval instar, wL3), a wave of differentiation called morphogenetic furrow moves across the eye disc from posterior to anterior, promoting proliferation and differentiation of progenitors into photoreceptors. Photoreceptors organize in highly regular eye units, ommatidia, forming the adult compound eye (Baker, 2001; Ready et al., 1976; Figure 1A). Using the UAS-Gal4 system (Brand and Perrimon, 1993), we overexpressed *Drosophila* MYC (Myc) in undifferentiated eye imaginal disc progenitors by inducing a *UAS-Myc* transgene with the *eyeless-Gal4* (*ey-Gal4*) driver (Halder et al., 1998; Hauck et al., 1999). Expression of *ey-Gal4* was confirmed using a *UAS-dGFP* reporter, which encodes a rapid turnover (destabilized) GFP for sensitive detection of transient gene expression (Li et al., 1998; Supplementary Figure S1). In these conditions, a twofold increase in Myc transcripts is detected by qPCR analyses, while *Drosophila* Max (Max) remains unchanged, at least at the transcript level (Supplementary Figure S2). Notably, 75% of the animals show morphological defects, which can be grouped in two phenotypic classes based on the extent and intensity of the eye abnormalities: high-Myc Class 1 - mild misalignment of the ommatidia confined in the posterior part of the eye (Supplementary Figure S3) or, in some cases, throughout the midline of the eye; high-Myc Class 2 – cone-shaped eye (Figures 1B–D’). To assess whether Myc overexpression can affect eye development in a dose-dependent manner, we enhanced the activity of the UAS-Gal4 system by increasing the number of Gal4 and UAS transgenes carried by the flies and/or by raising the incubation temperature (mild activation at 24 °C; strong activation at 29 °C). Increasing the levels of Myc overexpression indeed exacerbates the eye defects, as flies carrying one transgene copy (*1x ey-Gal4, 1x UAS-Myc*) display more high-Myc Class 1 and high-Myc Class 2 eye defects when raised at 29 °C (Figure 1E). Flies with *2x ey-Gal4, 2x UAS-Myc* could not be analyzed as they die at larval stage, likely due to *eyeless* expression in a few neuronal progenitors in the developing brain (Hauck et al., 1999; Noveen et al., 2000). Importantly, similar eye phenotypes are observed upon overexpression of human MYCN (*UAS-MYCN*). Defects caused by either Myc or MYCN overexpression can be rescued by the presence of a *Myc* loss-of-function allele (*Myc* ^*G0354*^) (Figure 1E)). This indicates functional conservation between fly and human MYC proteins.

**FIGURE 1:**
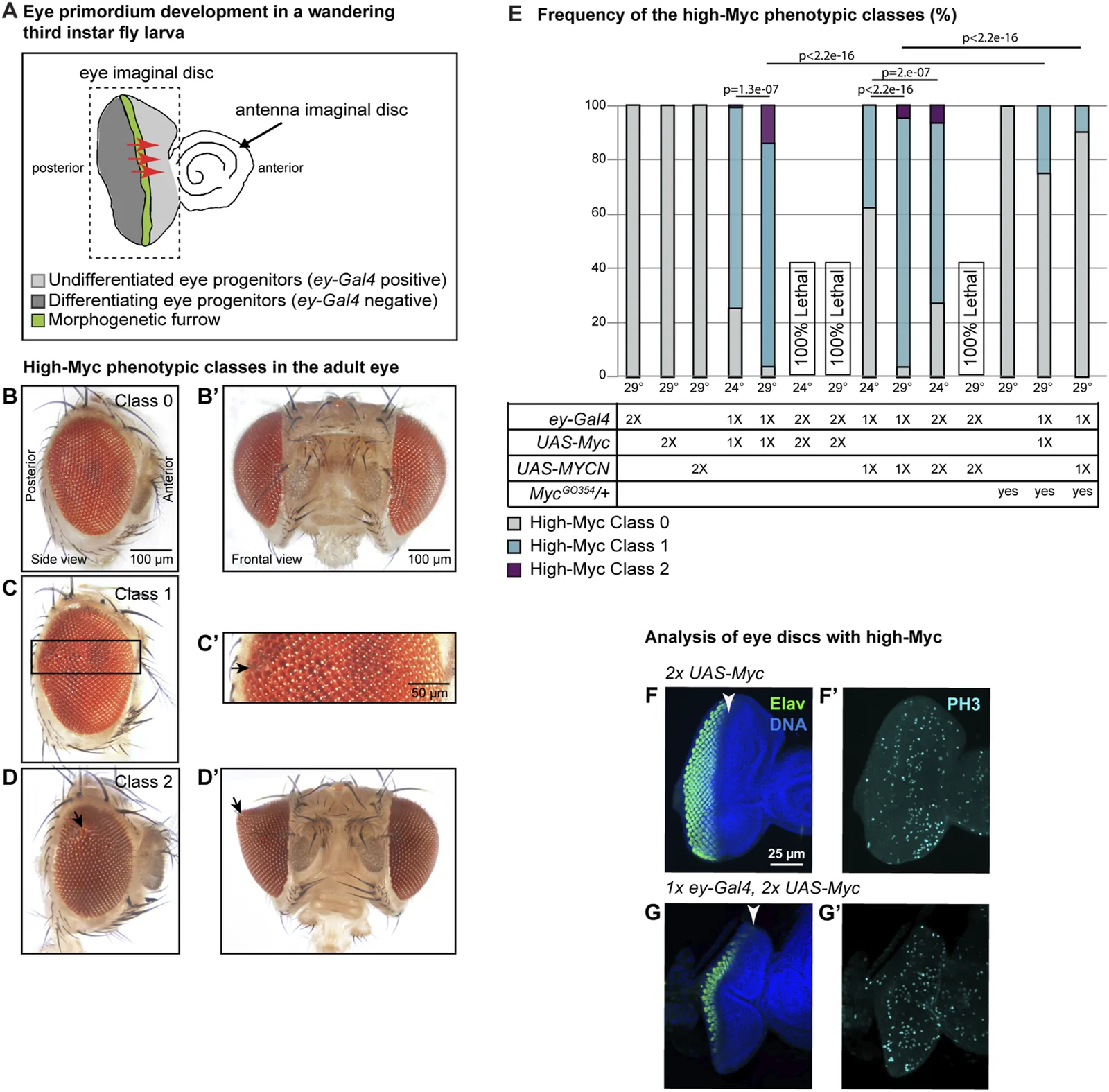
Overexpression of *Drosophila* Myc or human MYCN in eye progenitors disrupts eye development. **(A)** Schematic of the eye development in a wandering third instar (wL3) fly larva. The *Drosophila* eye develops from the larval eye imaginal disc, which expands through progenitor proliferation. At the third instar stage, the morphogenetic furrow sweeps from the posterior to the anterior eye disc margin (red arrows), triggering differentiation of photoreceptors, which will form the ~800 ommatidia (eye units) that make up the adult compound eye. In the developing disc, only undifferentiated progenitors express the *ey-Gal4* driver, which is not active in differentiating photoreceptors posterior to the morphogenetic furrow. **(B–D’)** Adult eye bright-field images showing the high-Myc phenotypic classes observed upon overexpressing *Drosophila* Myc (*UAS-Myc*) or the human MYCN (*UAS-MYCN*) in undifferentiated eye progenitors (*ey-Gal4*). Class 0: wild-type-like eyes **(B,B’)**; Class 1: mild ommatidial misalignment **(C,C’)**; Class 2: cone-shaped eyes. Arrows point to eye defects **(D,D’)**. The box in **(C)** indicates the magnified view in **(C’)**. **(E)** Quantification of the high-Myc phenotypic classes. Genotypes (1X = one transgene copy, 2X = two transgene copies) and rearing temperature are indicated below the bar chart. Of note, both Myc and MYCN overexpression phenotypes are rescued by heterozygous Myc mutation (*Myc* ^*GO354*^ */+*). N ≥ 150. Statistics: Mann-Whitney U test. **(F,G’)** Immunolabeling assay on eye discs from wL3 larvae upon Myc overexpression in undifferentiated eye progenitors. Differentiating photoreceptors are labeled with anti-Elav (green), proliferating cells with anti-PH3 (cyan) and nuclei with Hoechst (blue). Arrowheads mark the position of the morphogenetic furrow. The *2X UAS-Myc* genotype is shown as the control; however, the *ey-Gal4* control also does not display any eye-disc developmental abnormality (Supplementary Figure S1).

Defective differentiation and arrangement of ommatidia in the adult eye can arise from alterations during eye disc development (Baker et al., 1990; Baonza et al., 2001). To investigate this point, we performed immunolabeling assays on wL3 eye discs overexpressing Myc. The eye discs appear smaller, raising the possibility of altered proliferation, which could not be conclusively assessed by qualitative immunolabeling analysis with the mitotic marker phosphorylated Histone H3 (PH3) (Pérez-Cadahía et al., 2009). Interestingly, the number of cells positive for Elav, normally expressed in the differentiating photoreceptors posterior to the morphogenetic furrow (Robinow and White, 1991; Robinow and White, 1988), is reduced, with in some cases only a few differentiating cells remaining in the disc (Figures 1F,G’).

To summarize, Myc overexpression impinges on eye cell differentiation, leading to morphological alterations of adult eyes.

### Fly Max is required in undifferentiated eye progenitors to ensure tissue growth

To explore whether Max, like Myc, contributes to cell differentiation control, we downregulated Max in the undifferentiated eye progenitors using an RNAi construct targeting Max (*UAS-Max-RNAi*, hereafter *Max-RNAi*) driven by *ey-Gal4*. This strategy reduces the expression level of Max without affecting Myc transcripts, as assessed by qPCR analyses (Supplementary Figure S2). Consistent with the known growth-promoting function of Myc-Max dimers in physiological and tumoral contexts, downregulation of Max induces growth-defective eyes, while overall eye differentiation remains preserved (Figures 2A–C). The morphological defects fall in two phenotypic classes of increasing severity: low-Max Class 1 - showing mild loss of ommatidia; low-Max Class 2 - characterized by a significantly reduced eye size (Figures 2A–C). Introducing an extra copy of the *Max* gene (*Max-rescue* construct (Steiger et al., 2008)) rescues the eye phenotype in *1x ey-Gal4, 1x Max-RNAi* animals. By contrast, enhancing UAS-Gal4 efficiency by increasing transgene copies or temperature worsens the eye phenotype and ultimately leads to lethality at the end of the pupal stage, as the imago (the adult fly) never emerges from the pupal case (*2x ey-Gal4, 2x Max-RNAi* flies raised at 29 °C) (Figure 2D). These animals show small/absent eyes and a reduced head capsule (Figures 2E,F), two structures both developing from the eye disc (Haynie and Bryant, 1986).

**FIGURE 2:**
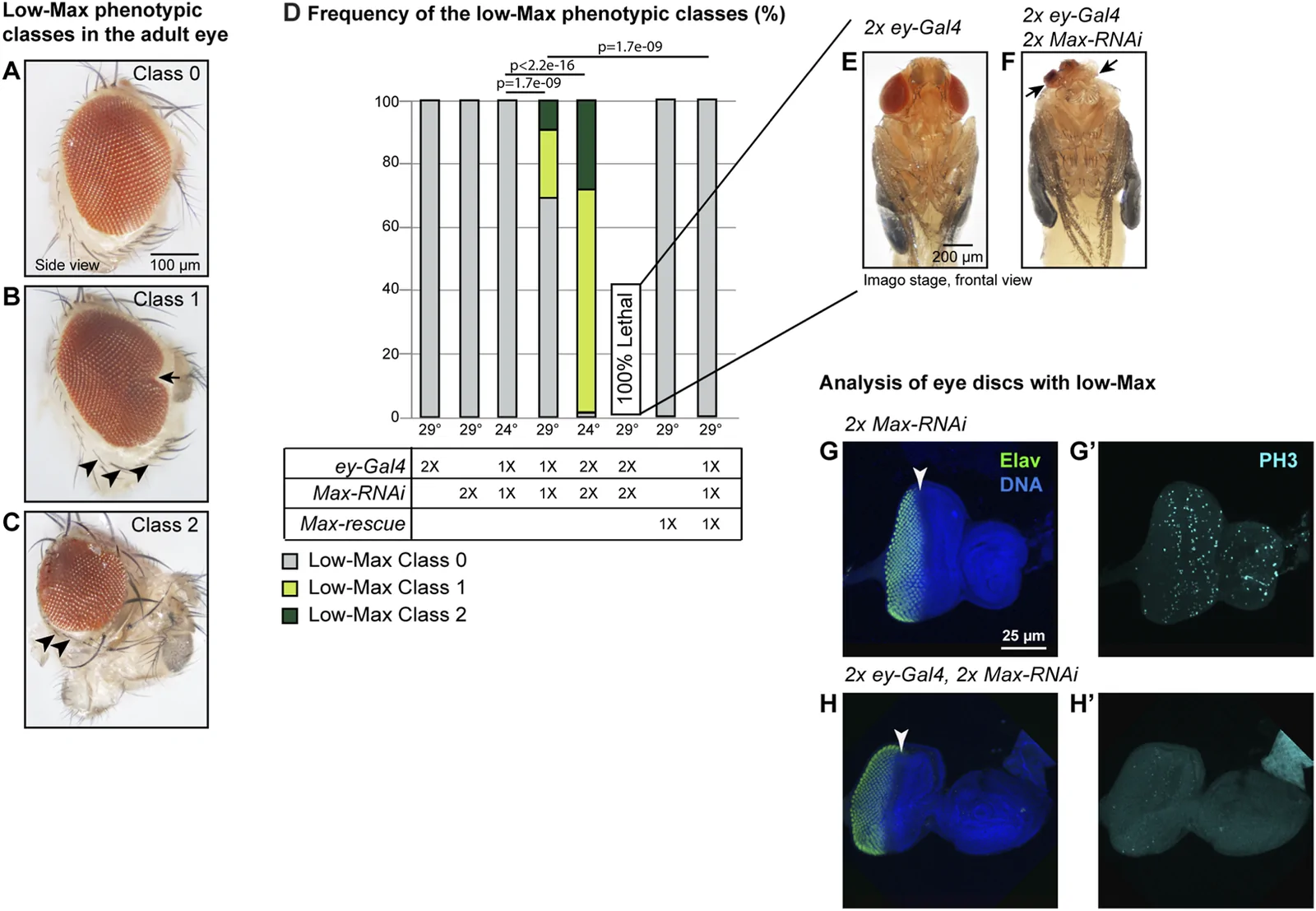
Downregulation of *Drosophila* Max in eye progenitors reduces eye growth. **(A–C)** Adult eye bright-field images showing the low-Max phenotypic classes observed upon downregulating *Drosophila* Max (*Max-RNAi*) in undifferentiated eye progenitors (*ey-Gal4*). Class 0: wild-type-like eyes **(A)**; Class 1: mild growth defect **(B)**; Class 2: small eye **(C)**. Arrows point to eye defects. Arrowheads indicate periorbital vibrissae, bristles that mark the perimeter of the adult eye territory. **(D)** Quantification of the low-Max phenotypic classes. Genotypes (1X = one transgene copy, 2X = two transgene copies) and rearing temperature are indicated below the bar chart. Of note, Max downregulation phenotype is rescued by an extra copy of Max (*Max-rescue*). N ≥ 130. Statistics: Mann-Whitney U test. **(E,F)** Bright-field images showing flies at imago stage upon Max downregulation in undifferentiated eye progenitors. Arrows point to eye and head defects. **(G–H’)** Immunolabeling assay on eye discs from wL3 larvae upon Max downregulation in undifferentiated eye progenitors. Differentiating photoreceptors are labeled with anti-Elav (green), proliferating cells with anti-PH3 (cyan) and nuclei with Hoechst (blue). Arrowheads mark the position of the morphogenetic furrow. The *2X Max-RNAi* genotype is shown as the control; however, the *ey-Gal4* control also does not display any eye-disc developmental abnormality (Supplementary Figure S1).

A small eye disc induced by Max downregulation could therefore account for the size-reduction in the adult eye and, in the most severe cases, adult head. Immunolabeling analyses on eye discs collected from larvae upon Max downregulation provide qualitative evidence supporting this hypothesis. Eye discs are smaller, and the signal from the proliferative marker PH3 weaker throughout the disc (Figures 2G,H’). Unlike upon Myc overexpression (Figures 1F,G’), cell differentiation is maintained upon Max silencing, as both the progression of the morphogenetic furrow and the appearance of Elav + differentiating photoreceptors are unaffected (Figures 2G,H’).

Altogether, these findings indicate that Max promotes tissue growth in the developing eye, yet it is dispensable for progenitor differentiation.

### Increasing Myc/Max ratio triggers cell transdetermination

Our data suggest that Myc regulates cell differentiation in a manner that does not strictly rely on Max, as high Myc expression, but not Max downregulation, impairs Elav expression (the classical marker of eye progenitor differentiation). Max-independent activities of Myc have been previously described in *Drosophila*, including the regulation of RNA polymerase III activity (Steiger et al., 2008). To further test whether Myc-mediated regulation of eye cell differentiation can occur independently of Max, we increased the intracellular Myc/Max ratio by simultaneously combining Myc overexpression with Max downregulation, hypothesizing that this would exacerbate the differentiation defects. Indeed, lowering Max levels would reduce the formation of Max-containing complexes, including Myc-Max dimers, while increasing the fraction of Myc not engaged in dimerization. Although effects arising from altered activity of Max-containing complexes cannot be excluded, this strategy would eventually increase the cellular pool of Myc available for functions that do not require Max. Hence, we generated flies carrying *ey-Gal4*, *UAS-Myc* and *Max-RNAi* transgenes. While 1*x ey-Gal4*, *1x UAS-Myc*, *1x Max-RNAi* flies raised at 24 °C are similar to controls, one-third of those raised at 29 °C exhibit a spectrum of mild phenotypes (mild high-Myc-low-Max Class 1, Figures 3A,B’), recapitulating the ommatidial loss (Figure 3B’ asterisks) observed upon Max downregulation (Figures 2A–C), and ommatidia misalignment (Figure 3B’ arrows) observed upon Myc overexpression (Figures 1B–D’), but not in Max-silenced eyes (please compare Figures 2B, 3B,B’).

**FIGURE 3:**
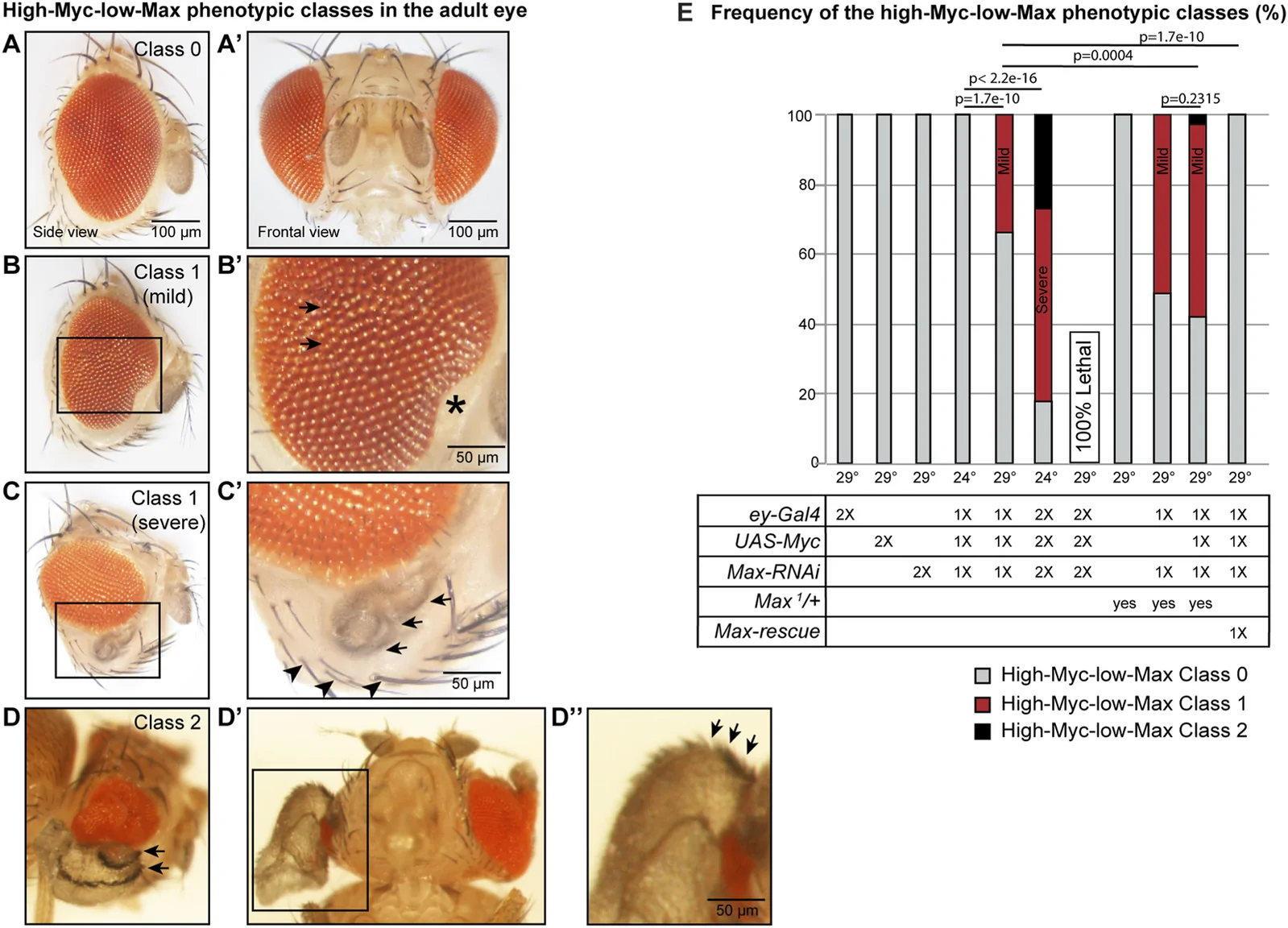
Enhancing Myc/Max ratio in eye progenitors induces eye-to-wing transdetermination **(A–D”)** Adult eye bright-field images showing the high-Myc-low-Max phenotypic classes observed upon overexpressing Myc (*UAS-Myc*) and downregulating Max (*Max-RNAi*) in undifferentiated eye progenitors (*ey-Gal4*), as well as upon strong but viable Max downregulation (*Max-RNAi* driven by *ey-Gal4* in animals carrying heterozygous *Max* ^*1*^ mutation). Class 0: wild-type-like eyes **(A,A’)**; Class 1: phenotypic spectrum ranging from mild cases in which some ommatidia are missing (asterisk) and others fail to properly align in the compound eye (arrows) **(B,B’)**, to severe cases in which ectopic, bristle-decorated tissue (arrows) develops in the eye territory delimited by periorbital vibrissae (arrowheads) **(C,C’)**; Class 2: wing-like structure in the eye territory characterized by bristles resembling those associated to the anterior wing margin (arrows) **(D–D”)**. **(E)** Quantification of the high-Myc-low-Max phenotypic classes. Genotypes (1x = one transgene copy, 2x = two transgene copies) and rearing temperature are indicated below the bar chart. *2x ey-Gal4*, *2x UAS-Myc*, *2x Max-RNAi* die when raised at 29 °C (likely due to Myc overexpression in few eyeless-expressing brain neuroblasts, similarly to *2x ey-Gal4*, *2x UAS-Myc.* Of note, high-Myc-low-Max phenotype is rescued by an extra copy of Max (*Max-rescue*)*.* N ≥ 200. Statistics: Mann-Whitney U test.

Importantly, increasing the number of transgene copies worsens the ommatidia phenotypes (Figure 3E). Indeed, 82% of *2x ey-Gal4*, *2x UAS-Myc*, *2x Max-RNAi* flies raised at 24 °C exhibit eye defects. 55% of those flies corresponds to severe high-Myc-low-Max Class 1 phenotypes (Figures 3C,C’) in which ventral ommatidia are replaced by ectopic, bristle-decorated tissue (Figure 3C’ arrows). This tissue still belongs to the eye territory as it lies internal to the periorbital vibrissae (Figure 3C’ arrowheads), bristles that mark the perimeter of the adult eye territory (Ouweneel, 1970; Haynie and Bryant, 1986). This suggests that part of the eye fails to differentiate into ommatidia and instead gives rise to other cell types, in contrast to the low-Max Class 2 phenotype (Figure 2C), where ommatidia are tightly surrounded by vibrissae, indicating a smaller but fully differentiated eye. Moreover, in 27% of cases, we observe a wing-like structure within the eye territory (high-Myc-low-Max Class 2, Figures 3D,E). The wing-like structure displays varying degrees of organization, ranging from a wing bud to a clearly recognizable, unfolded wing (Figure 3D”). In the latter case, they also contain sensory bristles characteristic of the anterior wing margin (Hartenstein and Posakony, 1989; Figures 3D,D” arrows). This could be assimilated to those observed in severe high-Myc-low-Max Class 1 flies (Figure 3C’ arrows), suggesting phenotypic continuity between high-Myc-low-Max Class 1 and high-Myc-low-Max Class 2.

The development of a wing-like structure in the eye represents a typical case of cell transdetermination, a process in which a cell switches fate and differentiates into another cell type. Importantly, no ectopic wing-like structures are observed when Myc overexpression nor Max downregulation are carried out separately, suggesting that the Myc/Max intracellular ratio more than their absolute levels accounts for Myc-mediated impact on cell fate and differentiation. To formally prove this point, we generated flies with low, yet viable Max levels by downregulating Max in heterozygous *Max* mutants (*1x ey-Gal4, 1x Max-RNAi, Max* ^*1*^ */+*). Strikingly, 51% of these flies show mild high-Myc-lox-Max Class 1 phenotypes. Further increase of the Myc/Max ratio by adding a *UAS-Myc* transgene (*1x ey-Gal41, 1x UAS-Myc, 1x Max-RNAi, Max* ^*1*^ */+*) leads to the appearance of high-Myc-low-Max Class 2 eyes and exacerbates the eye defects observed in *1x ey-Gal41, 1x UAS-Myc, 1x Max-RNAi* animals. Conversely, lowering the Myc/Max ratio by introducing an extra copy of the *Max* gene (*1x ey-Gal41, 1x UAS-Myc, 1x Max-RNAi, Max-rescue*) rescues the eye phenotype (Figure 3E).

Take together, these data demonstrate that the relative intracellular levels of Myc and Max regulate cell differentiation, with a high Myc/Max ratio triggering eye-to-wing transdetermination.

### The physiological HOX program is affected by high Myc/Max ratio

Cell identity is tightly regulated by HOX genes, evolutionarily conserved transcription factors whose misexpression is known to produce homeotic transformations, where one body part is converted into another (Emerald and Roy, 1997). The thoracic HOX gene *Antennapedia (Antp)* is normally expressed in the wing disc (Levine et al., 1983), where it antagonizes ectopic eye formation induced by the ectopic expression of the eye selector gene *eyeless*, which, together with other eye master regulators such as *twin of eyeless* and *sine oculis*, is responsible for eye development (Plaza et al., 2001). Ectopic Antp in the eye disc leads to an eye-to-wing transformation (Kurata et al., 2000; Prince et al., 2008; Gehring et al., 2009; Papadopoulos et al., 2011).

Given the above-mentioned phenotypes, we asked whether increasing Myc/Max ratio in eye progenitors results in ectopic expression of Antp. Remarkably, immunolabeling assays on eye disc of *2x ey-Gal4, 2x UAS-Myc, 2x Max-RNAi* wL3 larvae reveal the presence of Antp-expressing cells, which in extreme cases cluster together and associate with severe morphological defects of the eye disc (Figures 4A,B”). Few Antp + cells also express the neuronal marker Elav (Supplementary Figure S4A–B”), yet none of them express the mitotic marker PH3 (Figure 4B”; Supplementary Figure S4C,D”), suggesting that they have exited the cell cycle possibly to start differentiating. Importantly, Antp is also detected upon Myc, as well as MYCN, overexpression or strong Max downregulation (Supplementary Figure S5 A–D”’). Yet, in these cases Antp-expressing cells are scattered and positive for PH3, and the eye disc morphology is preserved (Supplementary Figure S5 A–D”’). Consistently, these combinations do not lead to a wing-like structure in the eye territory (Figures 1B–D’; Figure 3E). Together, these findings suggest that an increased Myc/Max ratio is sufficient to induce ectopic Antp expression in the eye disc and, in severe cases, activate a wing fate program.

**FIGURE 4:**
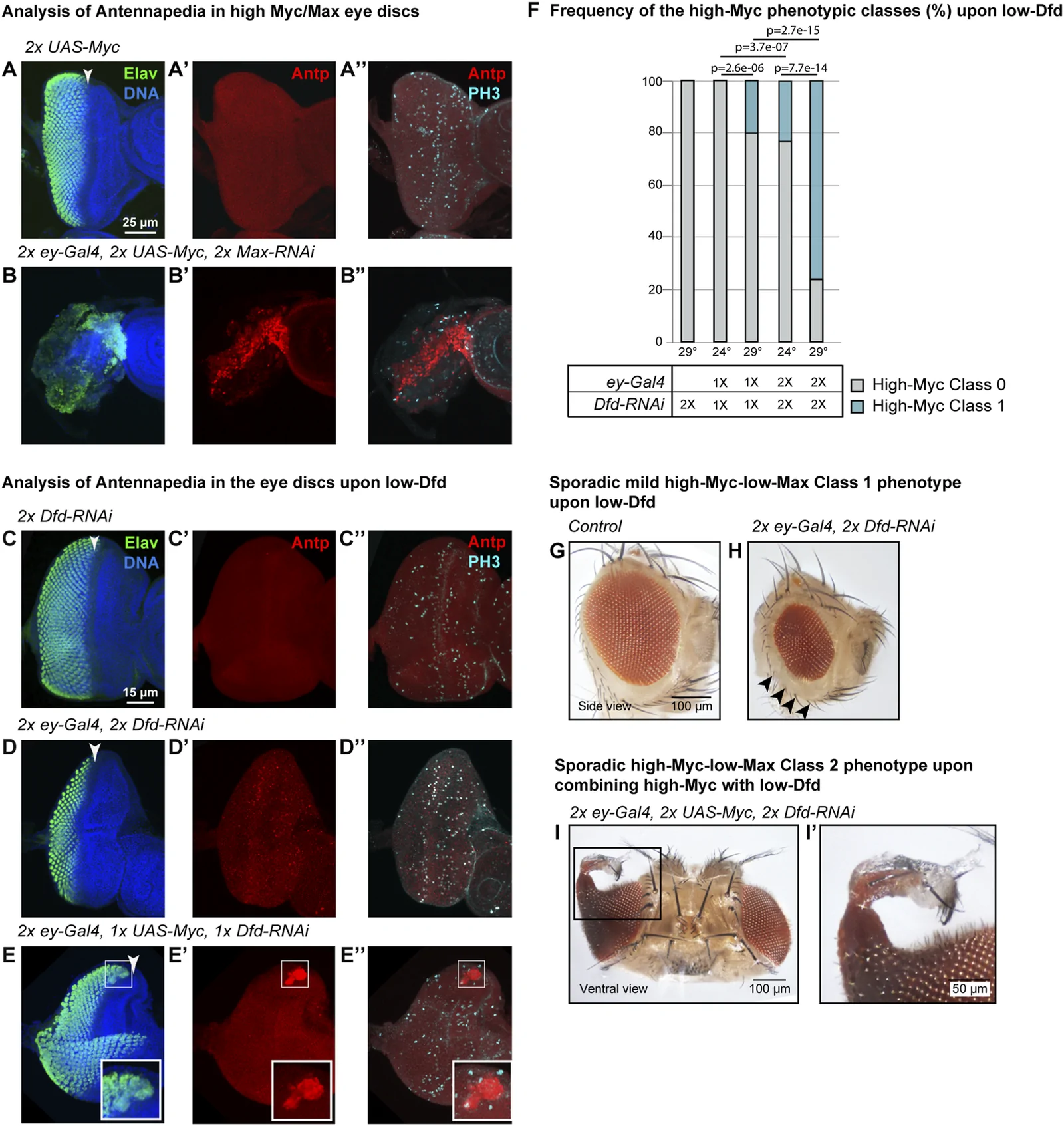
High Myc/Max ratio impacts the eye HOX program. **(A–E”)** Immunolabeling assay on eye discs from wL3 larvae upon **(A–B”)** Myc overexpression and Max downregulation, **(C–D”)** downregulation of Deformed (*Dfd-RNAi*), **(E–E”)** Myc overexpression and Dfd downregulation in undifferentiated eye progenitors (*ey-Gal4*). Differentiating photoreceptors are labeled with anti-Elav (green), Antennapedia positive cells with anti-Antp (red), proliferating cells with anti-PH3 (cyan) and nuclei with Hoechst (blue). Arrowheads mark the position of the morphogenetic furrow. **(F)** Quantification of the high-Myc phenotypic classes observed upon Dfd downregulation. Genotypes (1X = one transgene copy, 2X = two transgene copies) and rearing temperature are indicated below the bar chart. N ≥ 200. Statistics: Mann-Whitney U test. **(G,H)** Adult eye bright-field images showing mild high-Myc-low-Max Class 1 phenotype occasionally observed upon Dfd downregulation in undifferentiated eye progenitors **(H)** compared to a wild-type like eye of the same genotype **(G)**. Arrowheads indicate periorbital vibrissae. **(I,I’)** Adult eye bright-field images showing a wing-like structure (high-Myc-low-Max Class 2 phenotype) occasionally observed upon combining Dfd downregulation with Myc overexpression in undifferentiated eye progenitors.

Gain- or loss-of-function mutations in a Hox gene can alter segment identity by changing where that Hox gene itself is expressed and by disrupting the normal repression/activation interactions among neighboring Hox genes (Mallo and Alonso, 2013). The cephalic HOX gene *Deformed (Dfd)* is expressed in the eye disc (Diederich et al., 1991; Chadwick et al., 1990; Martinez-Arias et al., 1987), and *Dfd* loss-of-function mutations are known to result in transformations towards thoracic structures (Merrill et al., 1987; Regulski et al., 1987; Hughes and Kaufman, 2002). Being the wing a thoracic appendage, we speculated that ectopic Antp expression in the eye disc upon high Myc/Max ratio may be epistatic to Dfd. Dfd silencing in eye progenitors (*ey-Gal4, Dfd-RNAi*) is sufficient to drive ectopic expression of Antp in proliferating cells of the eye disc (Figures 4C,D”) and mimics Myc overexpression phenotypes in the adult eye in a dose-dependent fashion (Figure 4F). Interestingly, eye defects resembling mild high Myc-low-Max Class 1 (Figures 3B,C’) are also occasionally observed upon Dfd downregulation (Figures 4G,H), and they become more severe upon further Myc overexpression, supporting a genetic interaction between Dfd silencing and Myc overexpression. Indeed, *2x ey-Gal4*, *2x UAS-Myc, 2x Dfd-RNAi* flies in rare cases exhibit a tissue overgrowth bulging from the eye territory (Figures 4I,I’), resembling a rudimental wing-like structure similarly to what is induced by high Myc/Max ratio (Figure 3D,D”). Accordingly, the eye discs with Dfd downregulation and Myc overexpression occasionally show clustered, non-proliferating Antp + cells and disorganized Elav labeling (Figure 4E,E”), reminiscent of the phenotypes observed under high Myc/Max ratio (Figure 4B,B”).

Overall, these data demonstrate that loss of Dfd enables ectopic expression of Antp and synergizes with elevated Myc levels to induce cell transdetermination, revealing HOX genes as possible mediators of MYC-driven regulation of cell identity.

### High Myc/Max ratio globally disrupts the developing program of the eye progenitors

To gain a genome-wide and unbiased perspective on the molecular consequences of a high Myc/Max ratio in undifferentiated eye progenitors, we performed a bulk RNA sequencing (RNA-seq) assay on eye imaginal discs collected from *2x ey-Gal4, 2x UAS-Myc, 2x Max-RNAi* wL3 larvae raised at 24 °C or, as a control, from larvae with equal number of UAS and Gal4 transgenes (*2x ey-Gal4, 4x GFP-RNAi*). For each genotype, we performed three independent replicates, which group together in a Principal Component Analysis (Supplementary Figure S6) indicating consistency across replicates. Among the detected genes (Supplementary Table S1), we defined differentially expressed genes (DEGs) as those with |log2FC(high-Myc-low-Max/control)|>1, adjusted p-value < 0.05. A baseMean ≥ 14 (25th percentile cutoff) was used to exclude lowly expressed genes.

Out of the 835 DEGs identified, 314 genes are upregulated and 521 are downregulated (Supplementary Table S1). Interestingly, and in agreement with the observed phenotypes, gene ontology (GO) analysis of the downregulated genes highlights several eye development GO terms (Figure 5A; Supplementary Table S2). These include key eye regulators such as the Pax-family transcription factor Eyegone (Eyg), that promotes eye disc proliferation (Jang et al., 2003); the transcription factor Prospero (Pros) expressed in R7 photoreceptors (Kauffmann et al., 1996) and required for their differentiation (Cook et al., 2003); the transcription factor Retained (Retn) (Ditch et al., 2005), and the Heat shock protein 27 (Hsp27), whose downregulation affects ommatidia morphology producing rough eye phenotypes (Chen et al., 2012). Moreover, the RNA-binding protein Embryonic lethal abnormal vision (Elav) expressed in differentiating photoreceptors (Robinow and White, 1988) and whose mutation produce aberrant eye structures (Homyk et al., 1985) also shows a tendency to decrease (log2FC = −0.7) (Figure 5B). Overall, this indicates that a global suppression of the eye developmental program contributes to the morphological defects observed in adult eyes (Figures 3A–D”).

**FIGURE 5:**
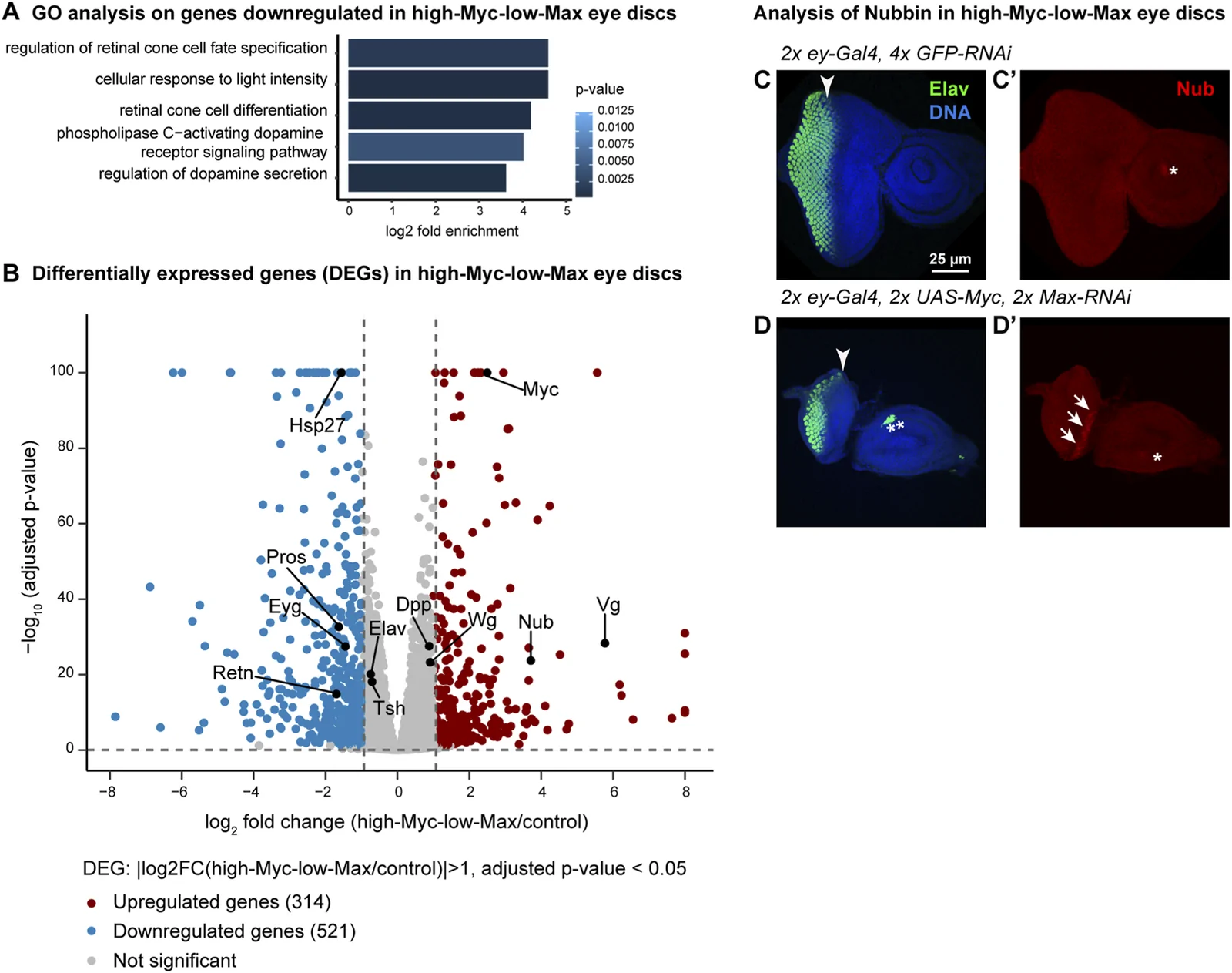
High Myc/Max ratio induces transcriptome-wide changes in the developing eye. **(A)** Gene ontology (GO) analysis on the downregulated genes identified in the bulk RNA-seq assay on high-Myc-low-Max (*2x ey-Gal4, 2x UAS-Myc, 2x Max-RNAi*) and control (*2x ey-Gal4, 4x GFP-RNAi*) wL3 eye discs (~120 h after egg lay at 24 °C). Differentially expressed genes (DEGs) were defined based on |log2FC(high-Myc-low-Max/control)|>1, adjusted p-value <0.05 and baseMean ≥14 (25th percentile cutoff). **(B)** Volcano plot of bulk RNA sequencing analysis. Each dot represents a gene. The 314 upregulated genes are shown in red, the 521 downregulated genes are shown in blue. Selected genes with roles in eye and wing development are labeled. **(C–D’)** Immunolabeling assay on eye discs from wL3 larvae upon Myc overexpression and Max downregulation in undifferentiated eye progenitors. Differentiating photoreceptors are labeled with anti-Elav (green), Nubbin positive cells with anti-Nub (red) and nuclei with Hoechst (blue). Arrowheads mark the position of the morphogenetic furrow. Arrows point to ectopic expression of Nub in the eye disc, whereas asterisks mark expected Nub expression in the antenna disc. Artifactual Elav expression in **(D)** is indicated by a double asterisk.

Importantly, wing development genes are upregulated in high-Myc-low-Max eye discs, including the transcription factors Vestigial (Vg), essential for wing identity as its mutation results in significantly reduced wings (Williams and Bell, 1988), and Nubbin (Nub), expressed in the nascent wing and required for proper proximo-distal wing patterning (Ng et al., 1995; Figure 5B). Two major interactors of Vg also show a tendency to increase (log2FC = 0.9): the TGFβ ligand Decapentaplegic (Dpp) (Mohit et al., 2006) and the WNT factor Wingless (Wg) (Klein and Arias, 1998; Baena-López and García-Bellido, 2003; Figure 5B), which create antero-posterior and dorso-ventral gradients essential for wing development and sensory organ formation (Neumann and Cohen, 1996; Nellen et al., 1996). Together with the proximo-distal information from Nub, these signals may provide a molecular basis for wing bud polarization, ultimately explaining the emergence of a wing-like structure with sensory bristles from the eye territory (Figure 3D”). Immunolabeling assays validate the ectopic Nub expression in high-Myc-low-Max eye discs (example shown in Figure 5C’,D’), as detected by our RNA-seq analysis.

In summary, a high Myc/Max ratio in undifferentiated eye progenitors impacts the HOX program and broadly suppresses the eye developmental cascade while ectopically activating a wing fate program.

## Discussion

The MYC family of transcription factors are well-known oncoproteins that, through dimerization with MAX, regulate key processes such as cell growth, proliferation and differentiation. However, in many cancers, MYC/MAX ratio is increased by MYC overexpression or MAX reduction (Dhanasekaran et al., 2022; Ferrucci et al., 2018; Bausch et al., 2017; Burnichon et al., 2012; Comino-Méndez et al., 2011), raising the possibility of a MAX-independent role of MYC. Using the *Drosophila* eye as an *in vivo* model, we identify MYC/MAX stoichiometry as a critical regulator of cell identity and differentiation. Elevated Myc levels relative to Max induces cell transdetermination culminating in an eye-to wing homeotic transformation. These findings reveal a role of Myc in controlling cell identity that is largely independent of Max, with broad implications for developmental biology and MYC-driven tumors.

### High Myc/Max ratio drives cell transdetermination while inducing a pro-oncogenic signature

Consistent with the role of Myc–Max dimers in promoting proliferation, Max downregulation in undifferentiated eye progenitors strongly reduces the size of the developing eye (eye imaginal disc), resulting in a small adult eye. By contrast, Myc overexpression in undifferentiated eye progenitors produces small eye discs with altered cell differentiation. This is likely not due to limiting Max, as a similar approach of Myc overexpression using the *GMR-Gal4* driver (expressed in differentiating eye cells) does increase cell size in the adult eye (Secombe et al., 2007) in a Max-dependent way (Steiger et al., 2008), indicating that Max levels are sufficient for Myc–Max function.

Myc overexpression may affect eye disc size through altered proliferation. Despite PH3 immunolabeling qualitative analysis did not reveal major changes in mitotic activity, a thorough quantification of PH3-positive cell density in the eye disc would be required to reliably detect proliferative defects. Increased cell death may also contribute to the reduced disc size. Bulk RNA-seq analysis does not support broad transcriptional activation of the apoptotic machinery: expression of the pro-apoptotic genes *grim*, *head involution defective (hid)* and *sickle (skl)*, as well as *Drosophila inhibitor of apoptosis protein 1 (DIAP1)*, the initiator caspase *Death regulator Nedd2-like caspase (Dronc)*, the effector caspases *Death-related ICE-like caspase (Drice)* and *Death caspase-1 (Dcp-1)*, and the apoptosome component *Death-associated APAF1-related killer (Dark)* (Xu et al., 2009), are not significantly altered (Supplementary Table S1). Nevertheless, because caspase activation is primarily regulated at the protein level, the contribution of apoptosis cannot be excluded and warrants further investigation.

Eventually, excess Myc in undifferentiated eye progenitors interferes with cell differentiation, producing cone-shaped eyes (high-Myc Class 2), suggesting that Myc functions in a context-dependent manner depending on whether it is overexpressed in undifferentiated cells (this study) or in cells already committed to terminal differentiation (Steiger et al., 2008). Increasing Max expression rescues the differentiation defects (Supplementary Figure S7), demonstrating that they arise from enhanced Myc/Max ratio, rather than absolute Myc levels. Moreover, further raising this ratio by combining Myc overexpression with Max silencing aggravates differentiation defects and induces transdetermination, converting eye progenitors into a wing-like structure.

Intriguingly, gene ontology (GO) analysis of genes upregulated in high-Myc/low-Max discs revealed enrichment of terms related to Myc’s oncogenic functions. This includes biological processes, such as trehalose metabolism, L-ornithine transport, and acetaldehyde metabolism (Supplementary Figure S8). Trehalose regulates organ size in flies by buffering glucose (Yasugi et al., 2017), and its levels increase in fly tumor models where Myc is upregulated (Prober and Edgar, 2002; Khezri et al., 2021). L-ornithine transport fuels polyamine synthesis, essential for cell proliferation in flies (Callaerts et al., 1992; Burnette and Zartman, 2015) and in mammals (Sánchez-Jiménez et al., 2019), as well as growth of MYC-associated tumors (Nilsson et al., 2005), including neuroblastoma (Gamble et al., 2021). Aldehyde dehydrogenases**,** key enzymes in the acetaldehyde metabolism, promote synthesis of polyamines and mark stem cell populations in several human cancers (Singh et al., 2013; Ginestier et al., 2007; Feldmann et al., 2007). This suggest that, although we did not observe overt tumoral growth in eye disc with elevated Myc/Max ratio, the pro-oncogenic cascade driven by high Myc may already begin to be activated.

Together, these findings indicate that Myc/Max stoichiometry governs cell growth and differentiation in a context-dependent manner, with a high Myc/Max ratio acting as a developmental switch promoting transdetermination of undifferentiated progenitors, while priming cells for tumorigenic transformation.

### High Myc/Max ratio impacts developmental programs and HOX gene expression

At the molecular level, the observed transdetermination resembles classical homeotic transformations and aligns with the role of HOX genes in maintaining segmental identity. Immunolabeling analyses revealed ectopic expression of the thoracic HOX gene *Antennapedia* (*Antp*) in high-Myc-low-Max eye discs, ranging from isolated Antp-expressing cells to Antp-positive clusters within morphologically altered tissue (Figures 4B,B”). This variability is consistent with the 27% penetrance of eye-to-wing transformation and likely caused Antp transcripts to fall below the detection threshold of bulk RNA sequencing. Nevertheless, our transcriptomic analysis identified a dual mechanism under high-Myc-low-Max conditions: (1) repression of eye differentiation genes (*eyegone, prospero, elav*), and (2) activation of wing determinants such as *nubbin* and *vestigial*, the latter being previously shown to be induced in the eye disc upon Antp overexpression (Kurata et al., 2000).

Multiple mechanisms may drive ectopic Antp expression. RNA sequencing data revealed downregulation of *teashirt* (*tsh*) (log2FC = −0.7, Figure 5A), a zinc finger transcription factor that cooperates with HOX proteins to define head identity by repressing Antp and whose loss induces ectopic Antp expression (Bhojwani et al., 1997). Because HOX domains are stabilized through mutual repression, we hypothesize that a Myc/Max ratio brought to the extreme also reduces expression of cephalic HOX genes such as *Deformed* (*Dfd*), which normally inhibits thoracic gene activity (Merrill et al., 1987; Regulski et al., 1987; Hughes and Kaufman, 2002). In agreement with this hypothesis, loss of Dfd phenocopies high-Myc-low-Max eye defects and synergizes with elevated Myc to enhance Antp activation and cell transdetermination, indicating genetic crosstalk between these HOX genes.

HOX gene rewiring (*Dfd* inhibition and *Antp* activation) and eye-to-wing developmental program shift could arise indirectly from altered promoter occupancy by residual Myc-Max dimers or from Max-independent Myc activity in the imaginal tissue. Localized apoptosis and associated ROS production can activate stress pathways, including TNF and JNK signaling, and promote compensatory proliferation that can facilitate cell-fate reprogramming in *Drosophila* imaginal discs (reviewed in (Worley and Hariharan, 2022)). A high Myc/Max ratio in eye progenitors could therefore promote transdetermination indirectly through such regenerative response. Our bulk RNA-seq analysis does not support broad transcriptional activation of the apoptotic (see above) or stress-response machinery. Key components of the JNK pathway, as the JNK ortholog *basket (bsk)* and its upstream and downstream regulators *hemipterous (hep)* and *puckered (puc)* (Ryoo et al., 2004; Lee et al., 2005; Santabárbara-Ruiz et al., 2015), the TNF pathway, including the ligand *eiger (egr)* and its receptors *grindelwald (grnd)* and *wengen (wgn)* (Fogarty et al., 2016), the ROS-responsive MAP kinase *p38a* (Santabárbara-Ruiz et al., 2015), and the regenerative factors *regeneration (reg)*, *augmenter of liver regeneration (alr)* and *lamina ancestor (lama)* (Klebes et al., 2005) are not significantly altered (Supplementary Table S1). Nevertheless, JNK targets as the transdetermination-associated mitogens *wingless (wg)* and *decapentaplegic (dpp)* (Maves and Schubiger, 1998; Ryoo et al., 2004), as well as the unpaired cytokines *upd1*, *upd2*, and *upd3*, which activate the JAK/STAT pathway required for regeneration, are upregulated upon increasing Myc/Max ratio (*wg* and *dpp*: log2FC = 0.9; *upd*, *upd2*, and *upd3*: log2FC > 3; p < 0.001; Supplementary Table S1). Together, these findings raise the possibility that Myc/Max imbalance may promote localized cell stress thereby engaging regenerative signaling and facilitating cell-fate reprogramming, a possibility that warrants further investigation.

A direct effect of Myc on chromatin and transcriptional regulation is also possible. Indeed, besides the well-recognized transcriptional activation role of MYC, which is MAX-dependent, MYC has also been shown to repress transcription, and this function does not necessarily require MYC–MAX dimers (Brenner et al., 2005; Gartel et al., 2001). Typically, in neuroblastoma, MYCN represses the TRKA and p75NTR neurotrophin receptors, whose robust expression predicts favorable prognosis, by binding their promoters and recruiting the histone deacetylase 1 (HDAC1) to establish a repressive chromatin state (Iraci et al., 2011). MYCN also recruit HDAC1 to represses tissue transglutaminase (TG2) and inhibit neuroblastoma cell differentiation (Liu et al., 2007). Similarly, high Myc may act on chromatin to repress Dfd, thus inducing ectopic Antp expression and cell transdetermination. In support of this model, the eye phenotypes observed upon Myc overexpression are partially rescued by heterozygous null mutations of dHDAC1 (*HDAC1* ^*04556/+*^) or the *Drosophila* homolog of human NCoR/SMRT, Smrter (*Smr* ^*G0060/+*^), that stabilizes HDAC recruitment into transcriptional repressor complexes (Mottis et al., 2013; Supplementary Figure S9). The downregulation of dHDAC1 or Smrter *via* RNAi in undifferentiated eye progenitors confirmed these results (Supplementary Figure S9). Despite providing promising genetic evidence, these findings do not establish a direct role of Myc in *Dfd* regulation, and the proposed chromatin-repression cascade remains a working hypothesis requiring future biochemical validation.

Collectively, these findings reveal that Myc/Max stoichiometry contributes to regulating the HOX network possibly through chromatin-repressive mechanisms, thereby safeguarding cellular identity.

### Pathological implications: a cancer perspective

Across 33 TCGA cancer types, approximately 28% of samples show MYC-family amplification (Schaub et al., 2018). Our *Drosophila* findings suggest that elevated MYC might contribute to cell-fate reprogramming as well as growth. If conserved in humans, this mechanism could potentially promote tumor heterogeneity, adaptation, progression, or treatment resistance (Scully et al., 2012; Fedele et al., 2024). In fly, the human MYCN reproduces *Drosophila* Myc phenotypes, including ectopic activation of the HOX gene *Antp*, supporting functional conservation but requiring validation in human models.

MYC-mediated chromatin remodeling may permit inappropriate HOX expression (Kress et al., 2015; Schaub et al., 2018; Parreno et al., 2022). In hematologic malignancies, MYC is associated with HOXA9 and HOXA10 upregulation (Li et al., 2019), while MYC–HOX cooperation may help maintain leukemic stem-cell identity and impaired differentiation (Miyamoto et al., 2021; Pelengaris and Khan, 2003). Thus, an altered MYC/MAX ratio might modulate a putative MYC–HOX circuit affecting cell-fate plasticity and tumor behavior. Restoring this balance could therefore merit investigation as a differentiation-promoting strategy.

### Evolutionary implications

Beyond its developmental relevance, MYC/MAX stoichiometry might have contributed to metazoan diversification. MYC and MAX are conserved regulators of growth, metabolism, and transcription (Young et al., 2011), and our findings raise the possibility that their balance could influence HOX expression and segmental identity. This would be consistent with the responsiveness of HOX boundaries to growth and metabolic signals (Soshnikova and Duboule, 2009). Because modest changes in HOX regulation are associated with morphological diversification (Carroll, 2008), evolutionary alterations in MYC dosage, MAX availability, or chromatin regulation might have contributed to developmental plasticity (Soshnikova and Duboule, 2009). MYC/MAX balance could therefore represent one possible link between metabolic regulation and patterning during evolution at least in vertebrates and certain invertebrates where the MYC/MAX complex is expressed (Hong et al., 2016).

## Conclusion

In conclusion, this study discloses the MYC/MAX stoichiometry as a critical regulator of cell fate reprogramming, extending MYC’s role beyond mere growth control to the maintenance of cellular identity. This mechanism may contribute to the well-known cellular heterogeneity of MYC-driven cancers, which is difficult to study *in vivo* and may underlie therapeutic resistance.

### Limitations of the study

While RT-qPCR assays confirm that our genetic manipulations of Myc and Max produced a significant shift in their transcript levels, post-transcriptional regulation could further influence their local, cell-autonomous stoichiometry. In particular, Myc abundance is regulated by mechanisms including ubiquitin-dependent degradation and phosphorylation-dependent control of protein stability and turnover (Galletti et al., 2009). Future studies using endogenous tag-knock-in lines or quantitative immunofluorescence will be valuable for determining Myc and Max protein abundance and dynamics at single-cell resolution.

Despite the genetic and molecular evidence supporting an interaction between an elevated Myc/Max ratio and *Deformed* regulation, we were unable to directly detect a reduction of *Dfd* expression in high-Myc-low-Max eye discs, due to biological and technical constraints. In particular, the available anti-Deformed antibody provides weak and unreliable staining in imaginal discs, preventing a confident assessment of changes in protein levels. Moreover, *Dfd* is expressed in a very restricted domain of the eye disc (Diederich et al., 1991; Chadwick et al., 1990; Martinez-Arias et al., 1987), making detection challenging and placing it below the sensitivity threshold of bulk RNA-seq. Finally, the incomplete penetrance and variable expressivity of the high-Myc-low-Max phenotype, together with fact that only a subset of cells within affected discs may undergo relevant changes, likely hindered our ability to quantify Dfd levels using quantitative PCR assay, as not all discs are equally and strongly affected. These limitations are likely to extend more broadly to our transcriptomic analysis. Because RNA-seq was performed on whole eye discs, genes expressed by restricted cell populations may remain undetected, and transcriptional changes specific to cells undergoing transdetermination may be diluted by unaffected cells. Therefore, we cannot exclude that the transcriptional impact of an elevated Myc/Max ratio in eye progenitors extends beyond what could be detected by our bulk RNA-seq analysis.

## Data Availability

The datasets presented in this study can be found in online repositories. The names of the repository/repositories and accession number(s) can be found below: Gene Expression Omnibus (GEO) under accession number GSE314316, https://www.ncbi.nlm.nih.gov/geo/query/acc.cgi?acc=GSE314316.
