# Supplementary material for: MYC/MAX balance dictates cell progenitor fate by altering the HOX program in the Drosophila eye
Source: Front Cell Dev Biol. 2026 Oct 26;14:1958701. doi: 10.3389/fcell.2026.1958701 (PMC13745702; doi:10.3389/fcell.2026.1958701)
Supplement: Supplementary file 1 [file DataSheet1.pdf]

Supplementary Figures

Supplementary Figure 1

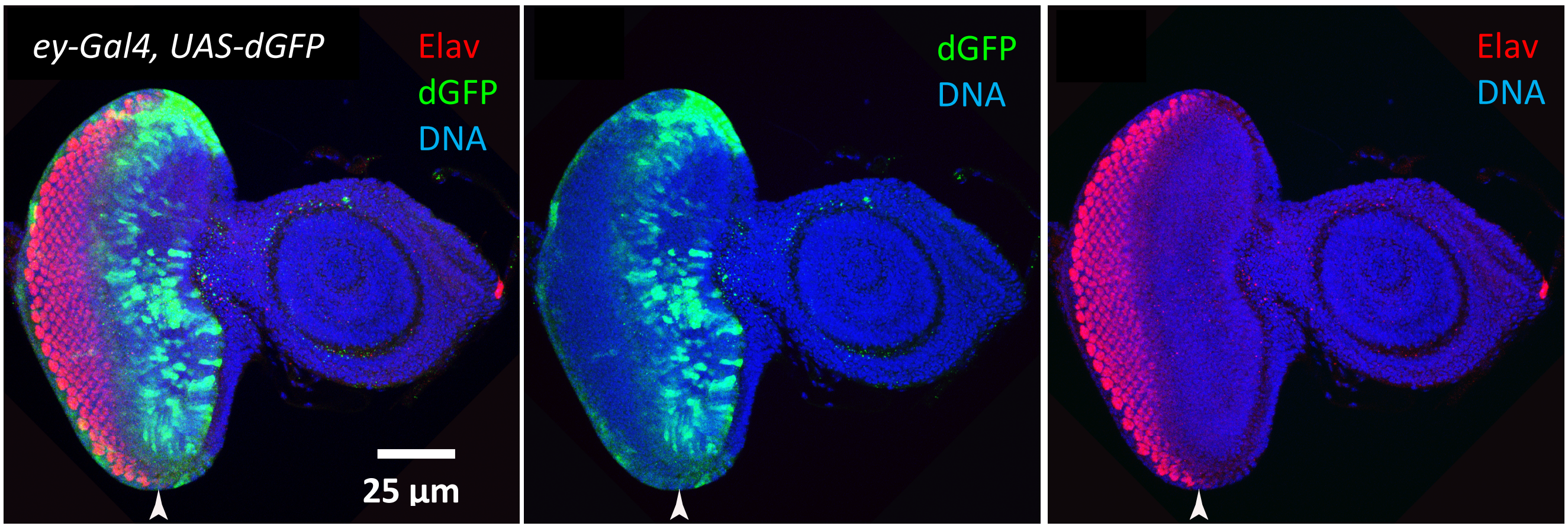

Supplementary Figure 1 - **The *ey*-Gal4 driver is expressed by undifferentiated progenitors in the eye disc located anteriorly to the morphogenetic furrow.**

Immunolabeling assay on eye discs from wandering third instar (wL3) larvae expressing a destabilized GFP (*UAS-dGFP*) under the control of the *eyeless* promoter (*ey-Gal4*). GFP is in green, differentiating photoreceptors are labeled with anti-Elav (red), and nuclei are labelled with Hoechst (blue). Arrowheads mark the position of the morphogenetic furrow.

Supplementary Figure 2

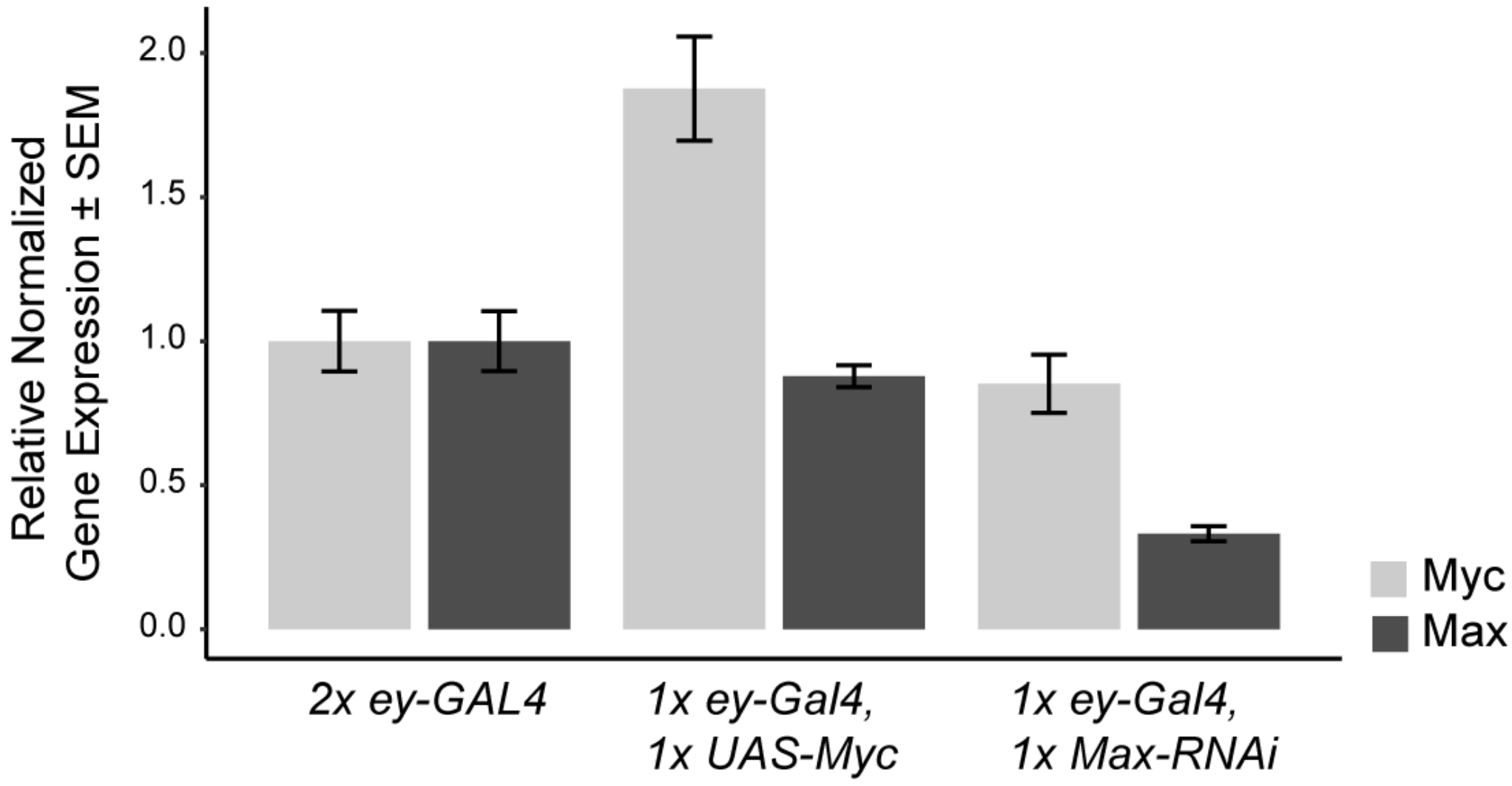

Supplementary Figure 2 – Quantification of Myc and Max levels following Myc overexpression and Max downregulation.

qPCR assay on eye discs from third instar wandering L3 larvae (wL3, ~90 hour after egg lay at 29°C) comparing Myc overexpression (*1x ey-Gal4, 1x UAS-Myc*) or Max downregulation (*1x ey-Gal4, 1x Max-RNAi*) to controls (*2x ey-Gal4*). Myc transcript levels approximately doubled in eye discs overexpressing Myc, while Max levels remained unchanged. Conversely, Myc levels were unaltered in *Max-RNAi* discs, whereas Max transcripts were markedly reduced. The bar chart shows the mean ± standard error mean (SEM) of two independent biological experiments (n = 2). Given the limited n, no statistical test was performed.

Supplementary Figure 3

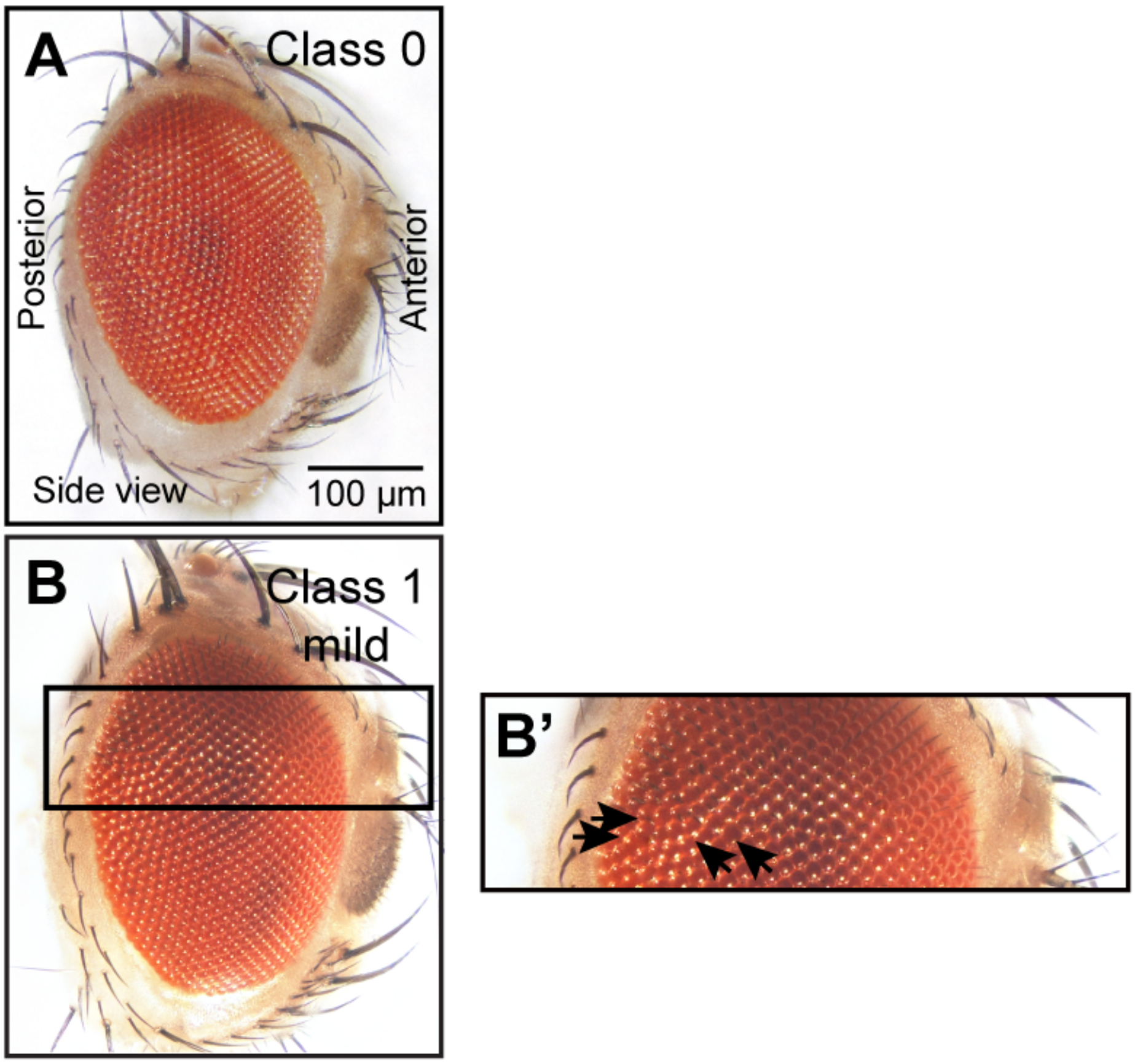

Supplementary Figure 3 – Mild high-Myc Class 1 phenotypic class in the adult eye.

Adult eye bright-field images showing mild high-Myc Class 1 phenotype observed upon overexpressing *Drosophila* Myc (*UAS-Myc*) or the human MYCN (*UAS-MYCN*) in undifferentiated eye progenitors (*ey-Gal4*) **(B)** versus a Class 0, wild-type-like eye **(A).** Arrows point to eye defects: ommatidia misalignment mostly located in the posterior part of the eye. The box in **(B)** indicates the magnified view in **(B')**.

Supplementary Figure 4

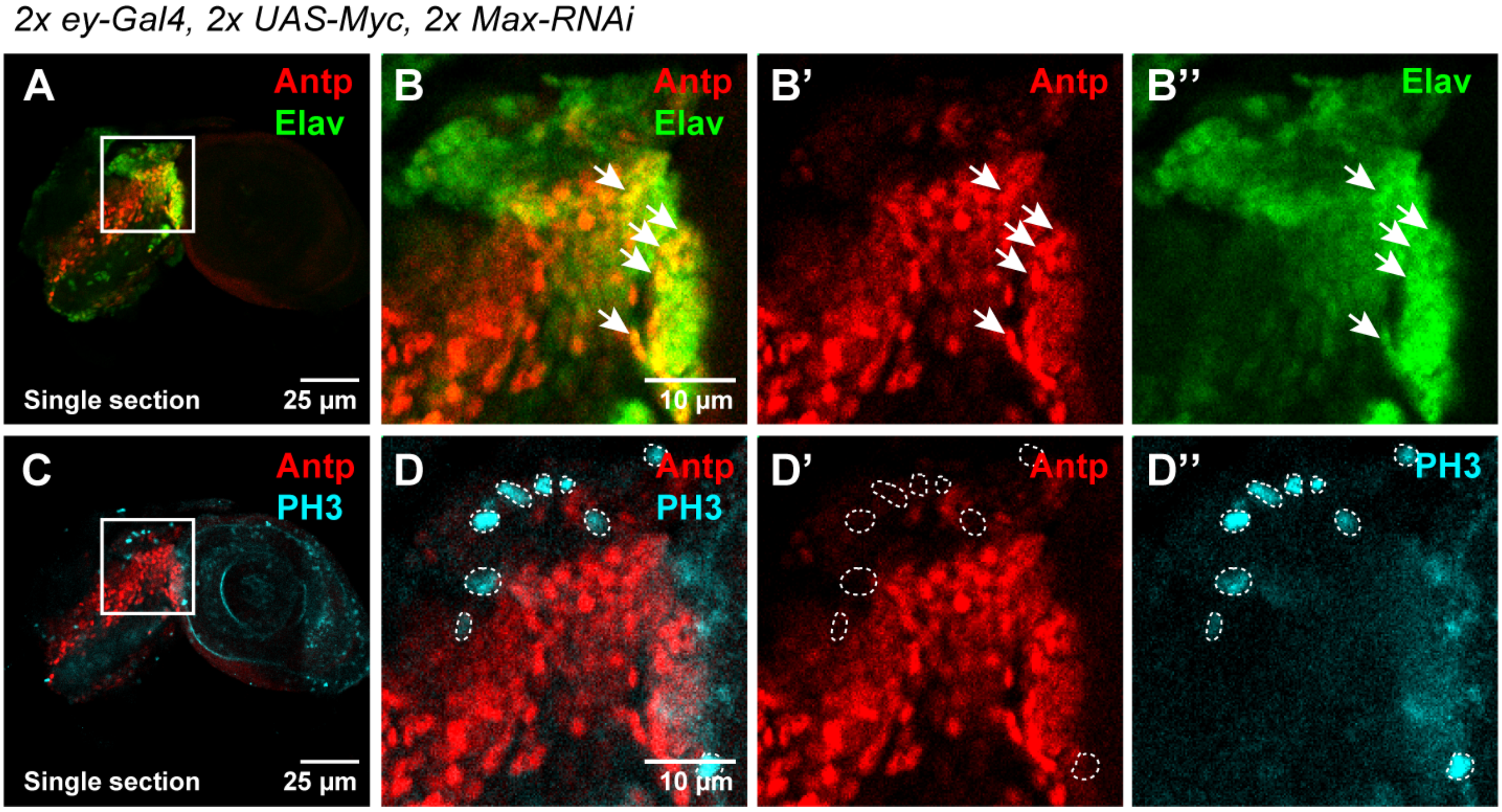

Supplementary Figure 4 – High Myc/Max ratio induces ectopic expression of the wing HOX gene *Antennapedia* (*Antp*) in both Elav⁺ and Elav⁻ non-mitotic cells of the eye disc.

Immunolabeling assay on eye discs from wandering third instar (wL3) larvae upon Myc overexpression and Max downregulation. **(A,C)** Single section confocal images of the eye disc shown in **Figure 4 B-B'''** with anti-Antp (red), anti-Elav (green) and anti-PH3 (cyan) labelling. **(B-B'')** High magnification of the region boxed in **(A)**, arrows indicate cells co-expressing Antp and Elav. **(D-D'')** High magnification of region boxed in **(C)** demonstrating absence of colocalization between Antp and PH3 signals.

Supplementary Figure 5

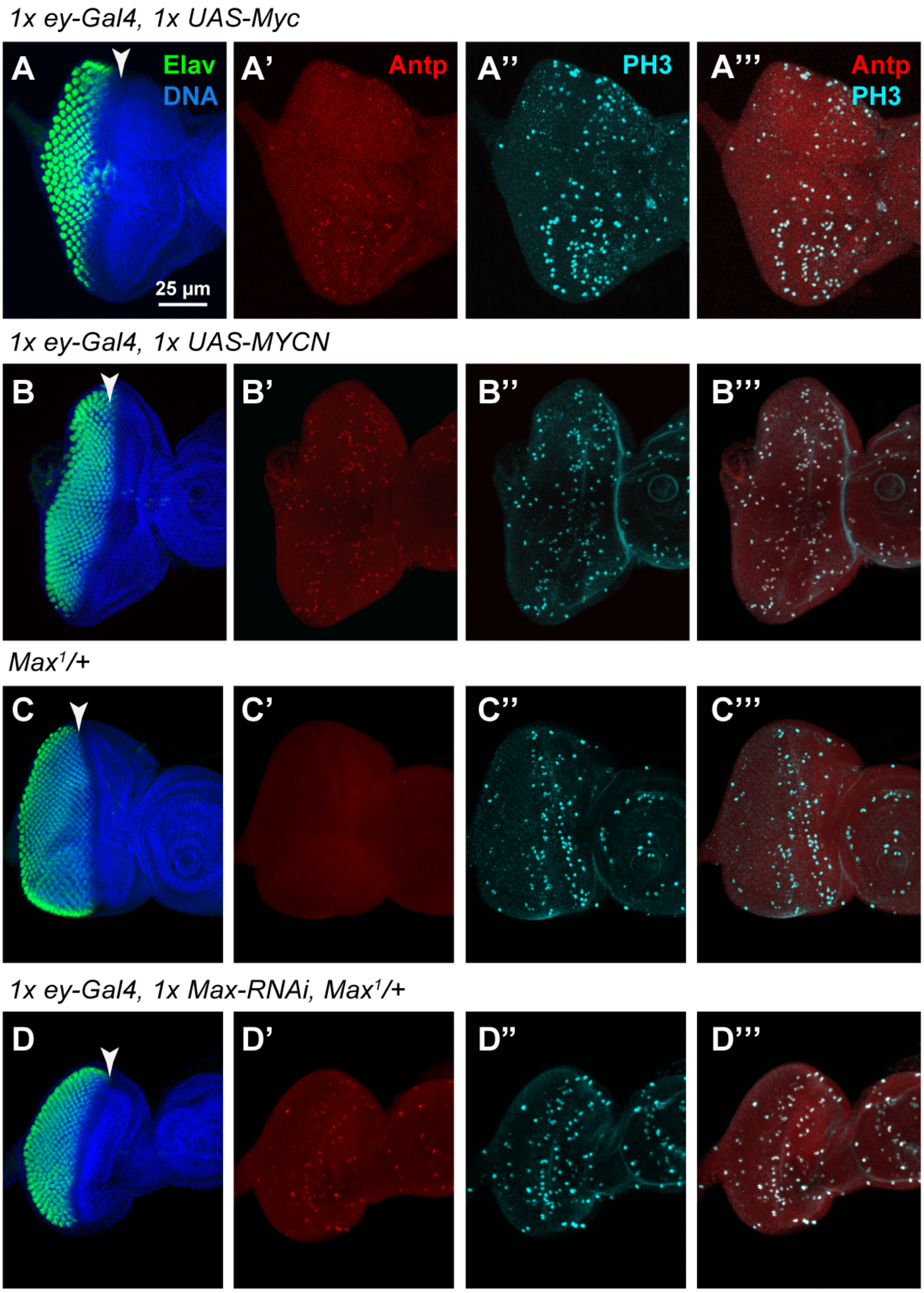

Supplementary Figure 5 - **The wing HOX gene *Antennapedia* (*Antp*) is ectopically expressed in eye discs upon high Myc/Max ratio.**

Immunolabeling assay on eye discs from wandering third instar (wL3) larvae upon **(A-A’’’)** Myc overexpression, **(B-B’’’)** human MYCN overexpression or **(D-D’’’)** strong but viable Max downregulation. Differentiating photoreceptors are labeled with anti-Elav (green), proliferating cells are labelled with anti-PH3 (cyan), anti-Antp is in red, and nuclei are labelled with Hoechst (blue). Arrowheads mark the position of the morphogenetic furrow.

Supplementary Figure 6

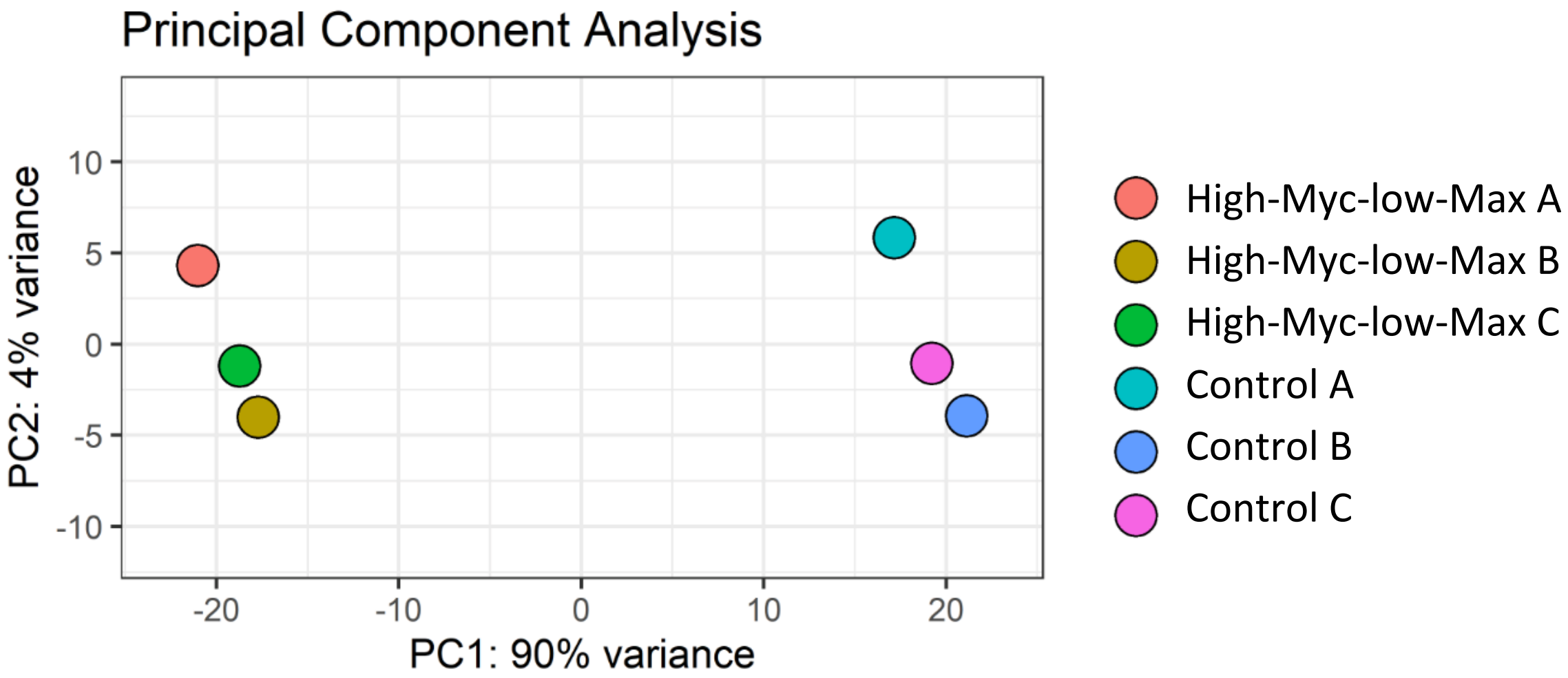

Supplementary Figure 6 - **Principal Component Analysis (PCA) of bulk RNA-seq data.**

Principal component analysis performed on the normalized expression profiles of all the samples of the bulk RNA-seq assay: *2x ey-Gal4, 2x UAS-Myc, 2x Max-RNAi* (High-Myc-low-Max) and *2x ey-Gal4, 4x GFP-RNAi* (Control) wL3 eye imaginal discs, each done in triplicate (A, B and C). Each point represents an individual sample. Principal component 1 (PC1) and principal component 2 (PC2) are shown on the x- and y-axes, and explain 90% and 4% of the total variance in the dataset. The plot illustrates clustering of samples by genotype, indicating consistency across replicates of the same genotype.

Supplementary Figure 7

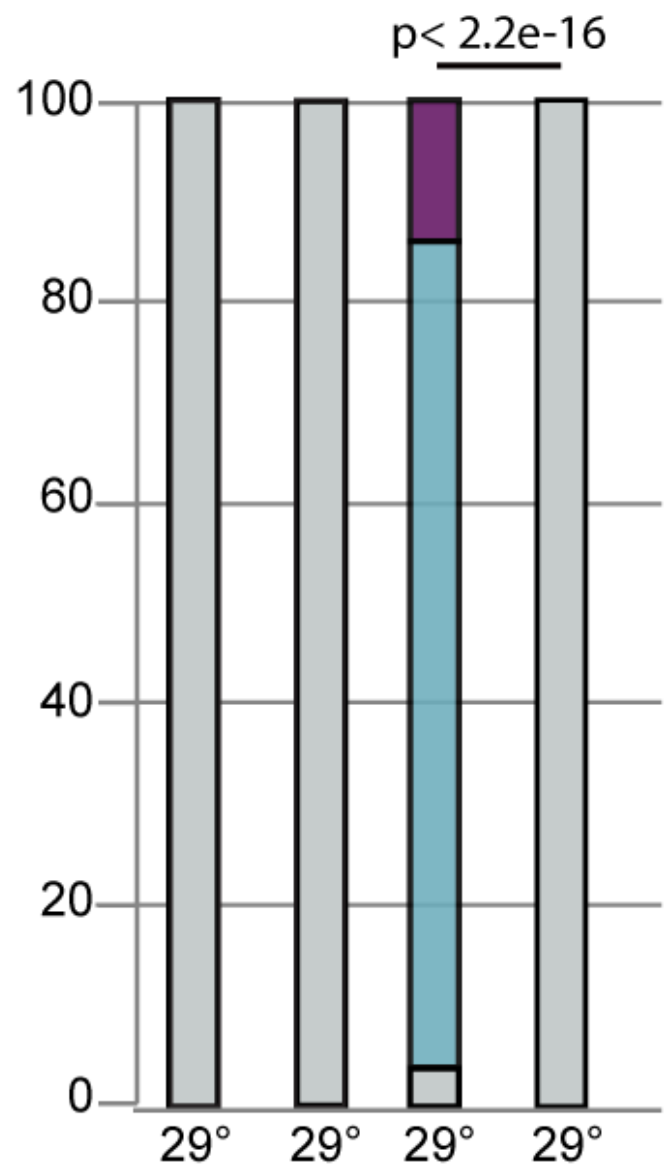

ey-Gal4	2X		1X	1X
UAS-Myc		2X	1X	1X
Max-rescue				1X

High-Myc Class 0
High-Myc Class 1
High-Myc Class 2

Supplementary Figure 7 – Increasing Max levels rescues the adult eye defects induced by Myc overexpression.

Quantification of the adult eye defects upon expressing the *UAS-Myc* and *Max-rescue* transgenes in the undifferentiated eye progenitors under the control of *ey-gal4*. Genotypes (1x = one transgene copy, 2x = two transgene copies) and rearing temperature are indicated below the bar chart. N ≥ 400. Statistics: Mann-Whitney U test.

Supplementary Figure 8

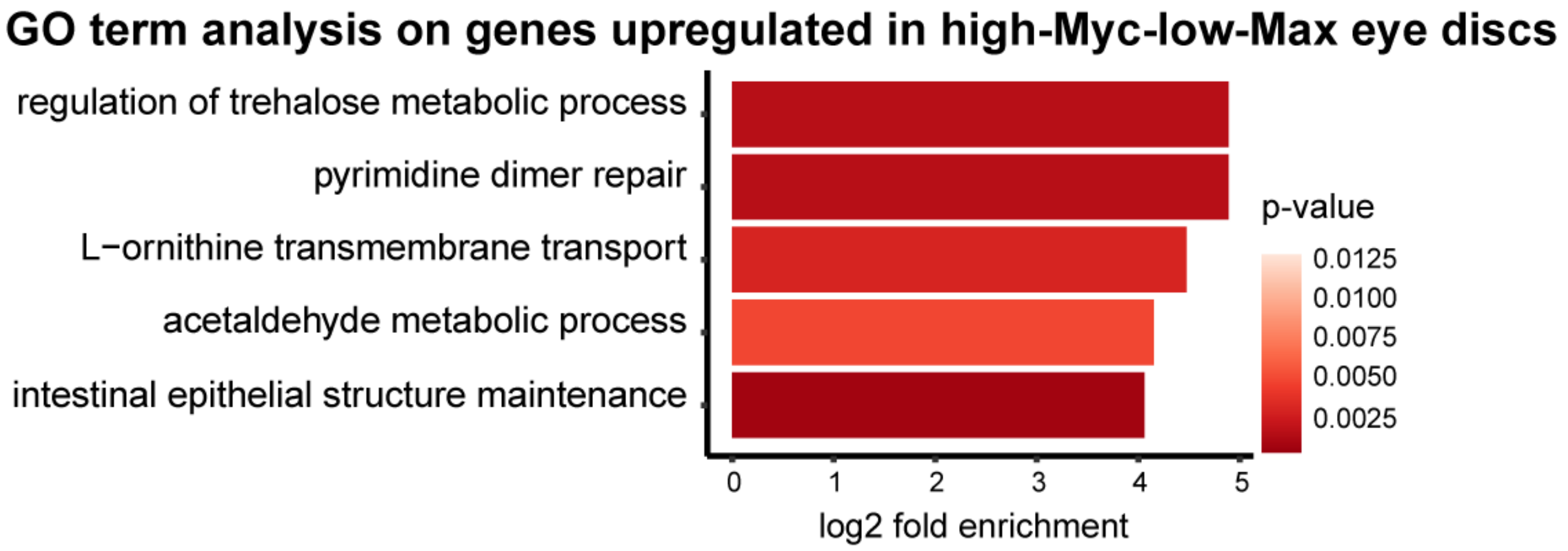

Supplementary Figure 8 – High Myc/Max ratio induces a pro-oncogenic signature in the developing eye.

Gene ontology (GO) analysis on the upregulated genes identified in the bulk RNA-seq assay on high-Myc-low-Max (*2x ey-Gal4, 2x UAS-Myc, 2x Max-RNAi*) and control (*2x ey-Gal4, 4x GFP-RNAi*) wL3 eye discs (~120 hour after egg lay at 24°C). Differentially expressed genes (DEGs) were defined based on |log2FC(high-Myc-low-Max/control)|>1, adjusted p-value < 0.05 and baseMean ≥ 14 (25th percentile cutoff). The top 5 GO terms are shown.

Supplementary Figure 9

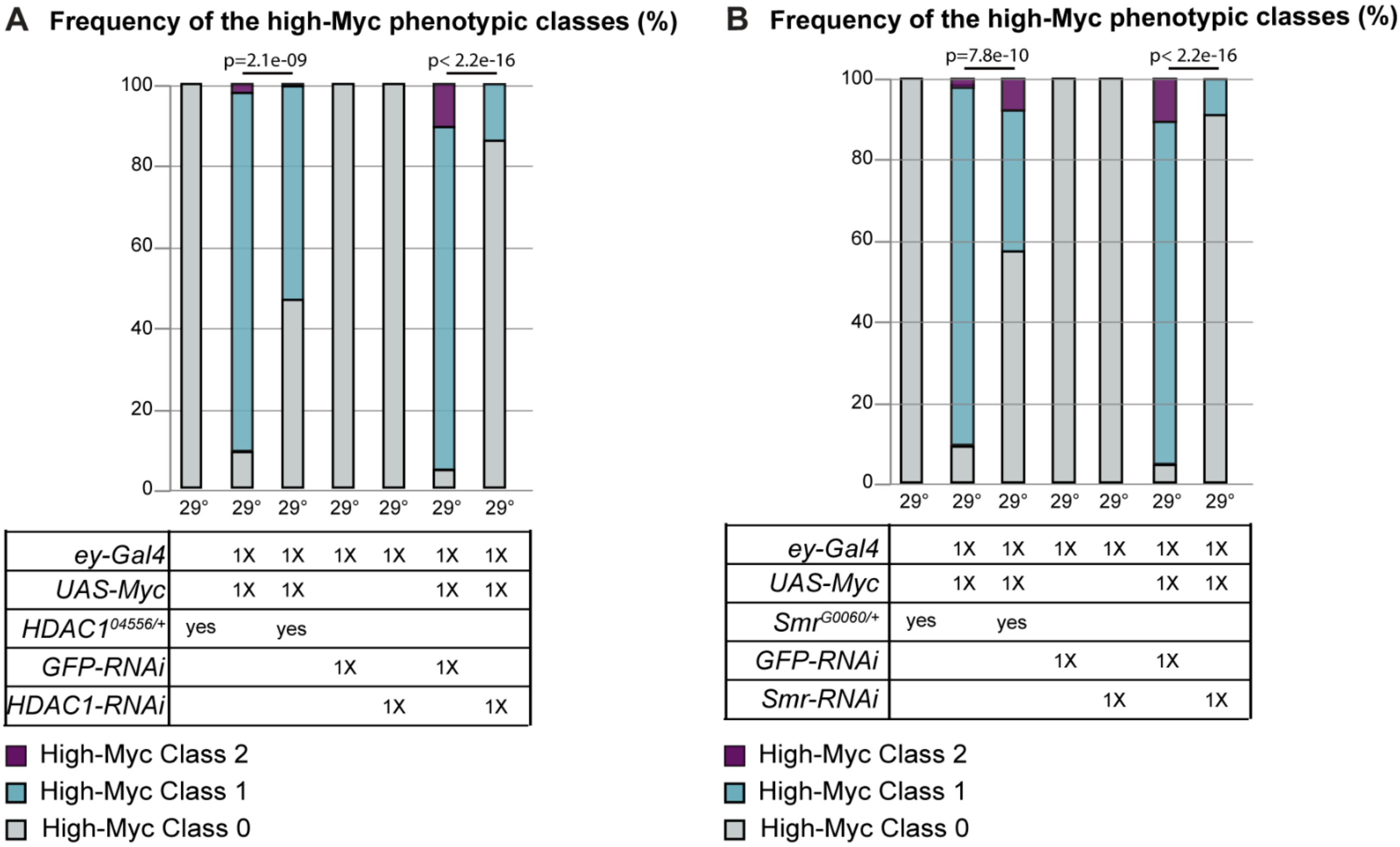

Supplementary Figure 9 – Lowering the levels of the Histone deacetylase 1 (HDAC1) or Smrter (Smr) transcriptional co-repressors rescues adult eye defects induced by Myc overexpression.

Quantification of the adult eye defects upon expressing *UAS-Myc* and silencing **(A)** HDAC1 or **(B)** Smr *via* RNAi in the undifferentiated eye progenitors under the control of *ey-gal4* or by using **(A)** HDAC1 or **(B)** Smr mutant alleles*.* Genotypes (1x = one transgene copy, 2x = two transgene copies) and rearing temperature are indicated below the bar chart. N ≥ 200. Statistics: Mann-Whitney U test.
